# Therapeutic targeting of pancreatic cancer stem cells by dexamethasone modulation of the MKP-1–JNK axis

**DOI:** 10.1074/jbc.RA120.015223

**Published:** 2021-01-13

**Authors:** Shuhei Suzuki, Masashi Okada, Tomomi Sanomachi, Keita Togashi, Shizuka Seino, Atsushi Sato, Masahiro Yamamoto, Chifumi Kitanaka

**Affiliations:** 1 Department of Molecular Cancer Science, Yamagata University School of Medicine, Yamagata, Japan; 2 Department of Clinical Oncology, Yamagata University School of Medicine, Yamagata, Japan; 3 Department of Ophthalmology and Visual Sciences, Yamagata University School of Medicine, Yamagata, Japan; 4 Department of Neurosurgery, Yamagata University School of Medicine, Yamagata, Japan; 5 Research Institute for Promotion of Medical Sciences, Faculty of Medicine, Yamagata University, Yamagata, Japan

**Keywords:** cancer stem cells, c-Jun N-terminal kinase (JNK), glucocorticoid receptor, anticancer drug, drug resistance, cancer-stem cells

## Abstract

Postoperative recurrence from microscopic residual disease must be prevented to cure intractable cancers, including pancreatic cancer. Key to this goal is the elimination of cancer stem cells (CSCs) endowed with tumor-initiating capacity and drug resistance. However, current therapeutic strategies capable of accomplishing this are insufficient. Using *in vitro* models of CSCs and *in vivo* models of tumor initiation in which CSCs give rise to xenograft tumors, we show that dexamethasone induces expression of MKP-1, a MAPK phosphatase, via glucocorticoid receptor activation, thereby inactivating JNK, which is required for self-renewal and tumor initiation by pancreatic CSCs as well as for their expression of survivin, an anti-apoptotic protein implicated in multidrug resistance. We also demonstrate that systemic administration of clinically relevant doses of dexamethasone together with gemcitabine prevents tumor formation by CSCs in a pancreatic cancer xenograft model. Our study thus provides preclinical evidence for the efficacy of dexamethasone as an adjuvant therapy to prevent postoperative recurrence in patients with pancreatic cancer.

Despite the overall improvement in cancer survival over the last few decades, most cancer patients today still succumb to the cancer they present with, and recurrence is a significant cause of cancer-related death (1, 2). Recurrence after seemingly successful initial treatment may limit the long-term survival of cancer patients and therefore may be a serious obstacle to a cure for cancer. Preventing recurrence, therefore, is considered essential to realizing the long-term survival of patients with cancer. Given their key role in tumor recurrence, tremendous efforts are being devoted to developing drugs that target cancer stem cells (CSCs), a subpopulation of cancer cells that can self-renew, initiate tumors, and resist conventional cancer therapies (2, 3). However, CSC-targeting drugs have not yet been successfully brought to clinical practice (4). Many factors, including safety, have made drug development challenging in general (5), but inadequate clinical trial design may require particular attention in the case of CSC-targeting drugs. The efficacy of drugs that specifically target CSCs may not be properly evaluated in clinical trials conducted on patients with advanced cancer for whom the growth of the tumor bulk, which consists mainly of non-CSCs, is the critical determinant of survival rather than recurrence from CSCs.

Pancreatic cancer is the seventh leading cause of cancer death worldwide (1). In the United States, the 5-year relative survival rate for pancreatic cancer is the lowest among major human cancers (6). Moreover, pancreatic cancer mortality is projected to be second only to lung cancer by 2030 (7). Hence, this malignant cancer will likely impose an increasing public health burden in the future. The highly intractable nature of pancreatic cancer is also demonstrated by its high probability of recurrence even after complete tumor resection (8), hence the need for novel therapeutic measures to prevent recurrence. Because CSCs have been identified in pancreatic cancer, a number of drugs that target molecules and pathways implicated in the control of pancreatic CSCs have been developed and advanced to clinical trials (9, 10); however, none have reached clinical practice.

Drug repurposing or repositioning is an unconventional approach to drug discovery. Repurposing explores new therapeutic benefits of existing drugs with known safety profiles, in contrast to developing drugs from scratch. Repurposing has a greater chance of success and can save both time and money because drug safety has already been validated (11, 12, 13, 14). Given the advantages of repurposing, we have been seeking to identify CSC-targeting drugs from among existing drugs with established safety profiles, irrespective of whether they have proven efficacy against cancer (15, 16, 17). In particular, we have focused on drugs that are being used safely in oncology practice for symptomatic treatment and have successfully identified anti-psychotic medications that have anti-CSC activities (18, 19, 20). While searching for CSC-targeting drugs, we identified the anti-CSC activities of dexamethasone, a synthetic glucocorticoid commonly used to treat patients with solid tumors intraoperatively and to treat cancer- and treatment-related symptoms. Importantly, the efficacy of dexamethasone in solid tumors has never been determined in clinical trials properly designed to evaluate drugs that specifically target CSCs.

## Results

### Dexamethasone inhibits CSCs from pancreatic cancer and other solid tumors by promoting their differentiation via glucocorticoid receptor activation

**Figure 1:**
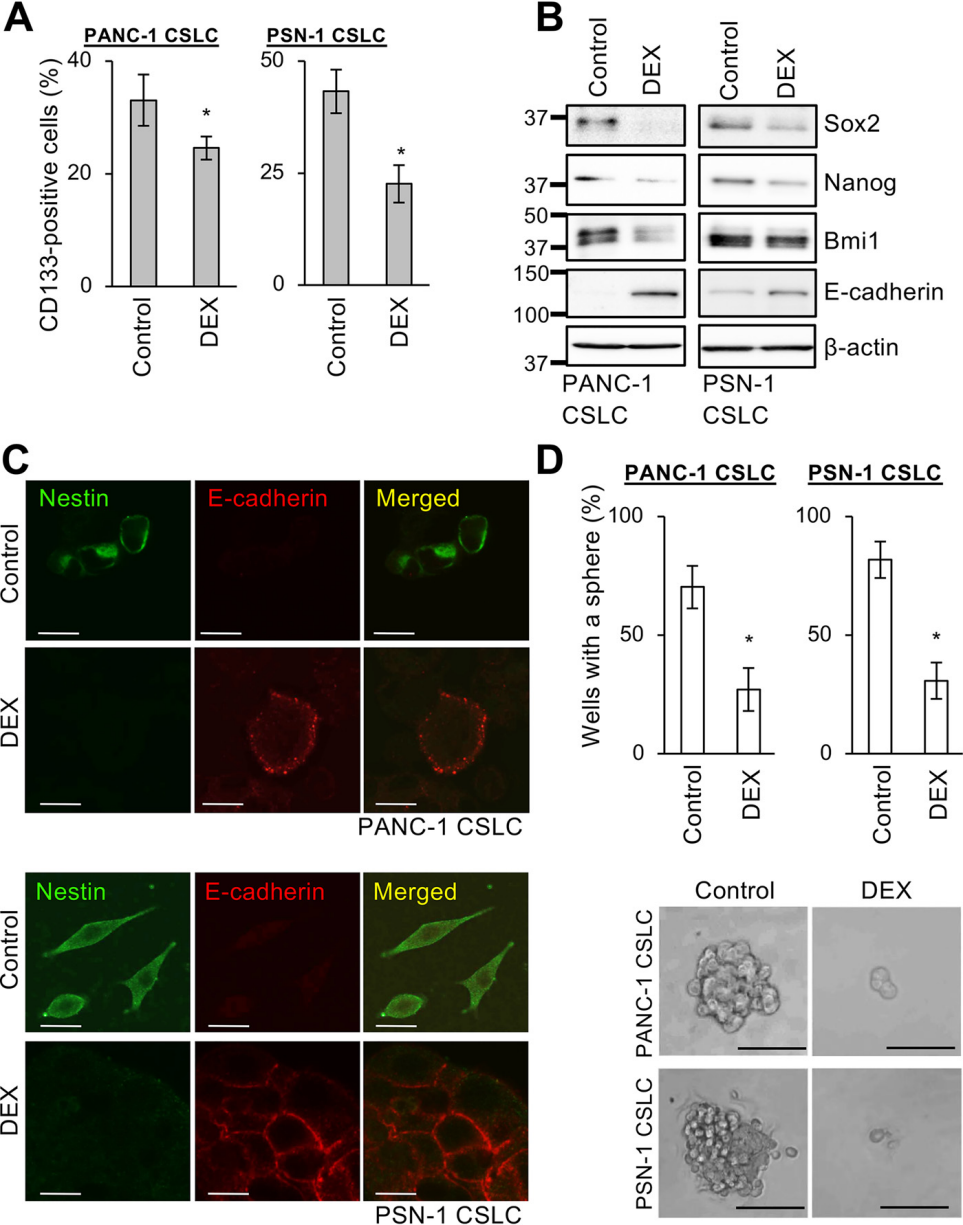
**Dexamethasone induces the loss of stemness and promotes differentiation of pancreatic CSCs.***A*, human pancreatic CSCs (PANC-1 CSLC and PSN-1 CSLC) cultured with either 1 μm DEX or without DEX (*Control*) for 6 days were subjected to flow cytometry to detect cell-surface expression of CD133; the percentage of CD133-positive cells was determined. Data are shown as mean ± S.D. from three independent experiments. *, *p* < 0.05. *B* and *C*, the indicated proteins were detected in cells cultured as described in *A* by either immunoblotting *B* or immunofluorescence *C*. *Scale bars*, 20 μm. *D*, sphere formation assay for cells cultured as described in *A* in the absence of DEX. *Top*, percentage of wells in which a tumorsphere was formed from a single cell. Data are shown as mean ± S.D. (*error bars*) from three independent experiments. *, *p* < 0.05. *Bottom*, photomicrographs of representative wells. *Scale bars*, 200 μm.

**Figure 2:**
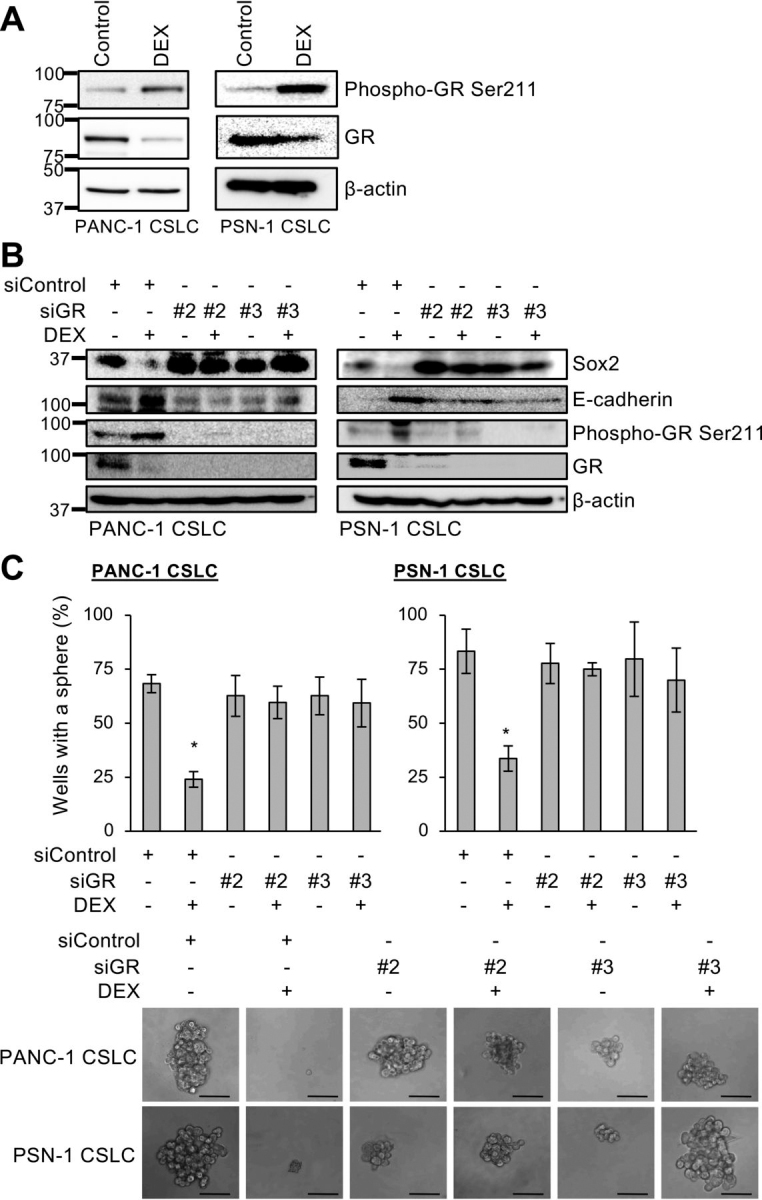
**Role of glucocorticoid receptor in dexamethasone-induced effects on pancreatic CSCs.***A*, levels of GR and GR phosphorylated at Ser-211 determined by immunoblotting in cells (PANC-1 CSLC and PSN-1 CSLC) cultured either with 1 μm DEX or without DEX (*Control*) for 6 days. *B*, cells transiently transfected with either siRNAs against the glucocorticoid receptor gene (*siGR*, #*2* and #*3*) or control siRNA (*siControl*) for 24 h were either treated with 1 μm DEX or left untreated for 6 days. The indicated protein levels were determined by immunoblotting. *C*, sphere formation assay for cells cultured as described in *B* in the absence of DEX. *Top*, percentage of wells in which a tumorsphere was formed from a single cell. Data are shown as mean ± S.D. (*error bars*) from three independent experiments. *, *p* < 0.05. *Bottom*, photomicrographs of representative wells. *Scale bars*, 200 μm.

To determine whether dexamethasone has anti-CSC activities, we used CSC lines from cancer types in which glucocorticoid receptor (GR) expression has been verified but the therapeutic efficacy of dexamethasone had yet to be demonstrated (21, 22, 23, 24, 25). We started with pancreatic cancer CSCs (PANC-1 CSLC and PSN-1 CSLC) and determined the effect of dexamethasone on the expression of stem cell–associated markers (CD133, Sox2, Nanog, Bmi1, and Nestin) and the differentiation marker E-cadherin. Treatment with dexamethasone at a concentration (1 μm) that was not toxic to normal cells (Fig. S1) uniformly reduced the expression of stem cell markers, whereas E-cadherin expression increased (Fig. 1, *A–C*). Significantly, exposure to dexamethasone for as long as 6 days was sufficient to cause the cells to commit fully to differentiation because Sox2 and E-cadherin markers continued to decrease and to increase, respectively, even in the absence of dexamethasone thereafter (Fig. S2). To determine whether the altered expression of stem cell and differentiation markers by CSCs is accompanied by reduced self-renewal capacity, we tested the sphere-forming ability of CSCs treated with dexamethasone for 6 days and subsequently cultured in a dexamethasone-free medium; sphere formation was reduced following dexamethasone treatment (Fig. 1*D*). Thus, our data suggest that dexamethasone effectively induces differentiation and the loss of stemness in pancreatic CSCs. We observed similar results with CSCs from other cancer types, namely A549 lung cancer cells and A2780 ovarian cancer cells (Fig. S3). Because prednisolone, another synthetic glucocorticoid, had similar effects on pancreatic CSCs (Fig. S4), we suspected the involvement of GR in the CSC-inhibitory effects of these synthetic glucocorticoids. After confirming that dexamethasone activates GR, as indicated by Ser-211 phosphorylation and phosphorylation-dependent down-regulation (Fig. 2*A*) (26, 27), we examined the effect of siRNA-mediated GR knockdown on dexamethasone's CSC-inhibitory effects. Whereas GR knockdown alone had no discernible effects on pancreatic CSCs, it blocked dexamethasone's inhibitory effects on these cells, as indicated by the lack of change in Sox2 expression, E-cadherin expression, and sphere formation (Fig. 2, *B* and *C*). Together, these data indicate that dexamethasone induces the loss of stemness and differentiation in CSCs in a GR-dependent manner.

### Dexamethasone-induced expression of MAPK phosphatase MKP-1/DUSP1 inhibits the JNK pathway and promotes pancreatic CSC differentiation

**Figure 3:**
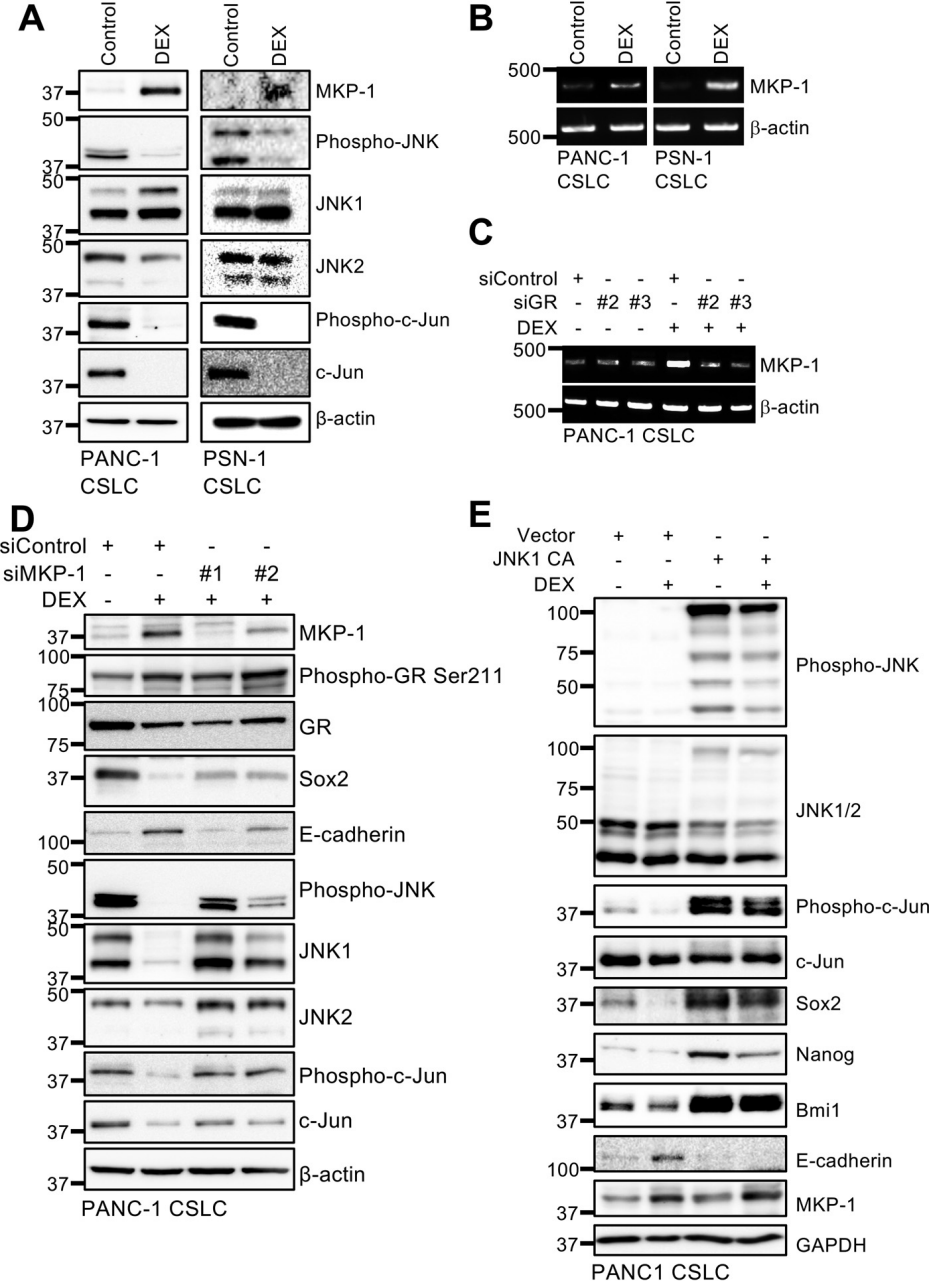
**Role of JNK inactivation by MKP-1 in dexamethasone-induced effects on pancreatic CSCs.***A* and *B*, the indicated proteins were detected by immunoblotting (*A*) and mRNAs were detected by RT-PCR (*B*) in cells cultured either with 1 μm DEX or without DEX (*Control*) for 6 days. *C*, PANC-1 CSLC cells transiently transfected with either siRNAs against the glucocorticoid receptor gene (*siGR*, #*2* and #*3*) or control siRNA (*siControl*) for 24 h were either treated with 1 μm DEX or left untreated for 6 days before RNA extraction for RT-PCR. *D*, PANC-1 CSLC cells transiently transfected with either siRNAs against MKP-1 (*siMKP-1*, #*1* and #*2*) or control siRNA (*siControl*) for 24 h were either treated with 1 μm DEX or left untreated for 6 days before immunoblotting for the indicated proteins. *E*, PANC-1 CSLC cells transiently transfected with either activated JNK1 expression plasmid (*JNK1 CA*) or empty control vector (*Vector*) for 24 h were either treated with 1 μm DEX or left untreated for 6 days. The indicated proteins were detected by immunoblotting.

In an attempt to elucidate the mechanism underlying dexamethasone's CSC-inhibitory effects, we examined molecular pathways implicated in regulating the stem cell state of pancreatic CSCs (9, 10, 28, 29). We observed reduced levels of phosphorylated JNK and c-Jun (Fig. 3*A*), which suggests that dexamethasone inhibits the JNK pathway. Because JNK is essential for maintaining the stemness of pancreatic CSCs, and JNK inhibition alone is sufficient to promote their differentiation (28, 29), this finding suggests that dexamethasone may inhibit CSCs by down-regulating the JNK pathway.

Apart from playing an essential role in CSC maintenance in a variety of human cancers, including pancreatic cancer (28, 29, 30, 31, 32, 33, 34), JNK is well known as a proinflammatory kinase (35). We therefore surmised that a dual-specificity MAPK phosphatase, MKP-1/DUSP1, a representative mediator of dexamethasone's anti-inflammatory effects (36), could be the link between dexamethasone and the JNK pathway in CSC regulation in pancreatic cancer. Consistent with this idea, dexamethasone induced MKP-1 protein expression in pancreatic CSCs (Fig. 3*A*), which was also observed in lung and ovarian CSCs (Fig. S3B). We also confirmed increased *MKP-1* mRNA expression after dexamethasone treatment (Fig. 3*B*) and the dependence of this increase on GR (Fig. 3*C*), which agrees with earlier studies that demonstrated glucocorticoid-responsive element–dependent activation of *MKP-1* transcription by dexamethasone (37, 38).

Next, we determined whether the induction of *MKP-1* expression is required for dexamethasone to inhibit pancreatic CSCs. *MKP-1* knockdown by siRNA attenuated dexamethasone's effects on phospho-c-Jun, Sox2, and E-cadherin levels (Fig. 3*D*), suggesting that MKP-1 mediates the inhibition of the JNK pathway and the stemness of pancreatic CSCs by dexamethasone. Significantly, MKP-1 is a multifunctional phosphatase that dephosphorylates and inactivates multiple members of the MAPK family, including JNK, p38 MAPK, and ERK (37, 38). Indeed, we observed reduced levels of phospho-ERK in addition to reduced levels of phospho-JNK following dexamethasone treatment of pancreatic CSCs (data not shown). We therefore determined using an MKK7-JNK1 fusion protein whether inactivation of JNK by MKP-1 is specifically required for dexamethasone to inhibit CSCs. We observed that in pancreatic CSCs expressing this activated JNK protein, dexamethasone failed to undergo changes associated with the loss of stemness (Fig. 3*E*). Altogether, these data are consistent with a model in which dexamethasone-activated GR in turn activates *MKP-1* transcription. The MKP-1 protein will dephosphorylate and inactivate JNK, thereby driving pancreatic CSC differentiation. Given that approximately 6 days of continuous dexamethasone treatment is required for pancreatic CSCs to commit fully to differentiation, we conducted a time course analysis to monitor molecular components along the GR**-**JNK axis in CSCs during and beyond this period (Fig. S5). Whereas phosphorylated GR and MKP-1 levels increased progressively, phosphorylated JNK and c-Jun levels eventually declined over the long term, fluctuating early on in a multiphasic manner accompanied by a transient increase around 4 days after dexamethasone treatment. E-cadherin levels increased similarly in a multiphasic manner preceded by an initial decline, albeit not entirely in parallel with phosphorylated JNK and c-Jun. As reported previously (39), the transient activation of the JNK pathway observed around 4 days was most likely associated with dexamethasone-induced activation of the transforming growth factor-β pathway, as indicated by the parallel increase in phospho-Smad2 levels (Fig. S5). Our data suggest that dexamethasone in the early phase of treatment may activate multiple MKP-1–dependent and –independent signaling pathways and that, therefore, a short-term dexamethasone treatment may possibly result in failure for pancreatic CSCs to commit fully to differentiation.

### Systemic dexamethasone eliminates CSCs within tumors formed by pancreatic CSCs

**Figure 4:**
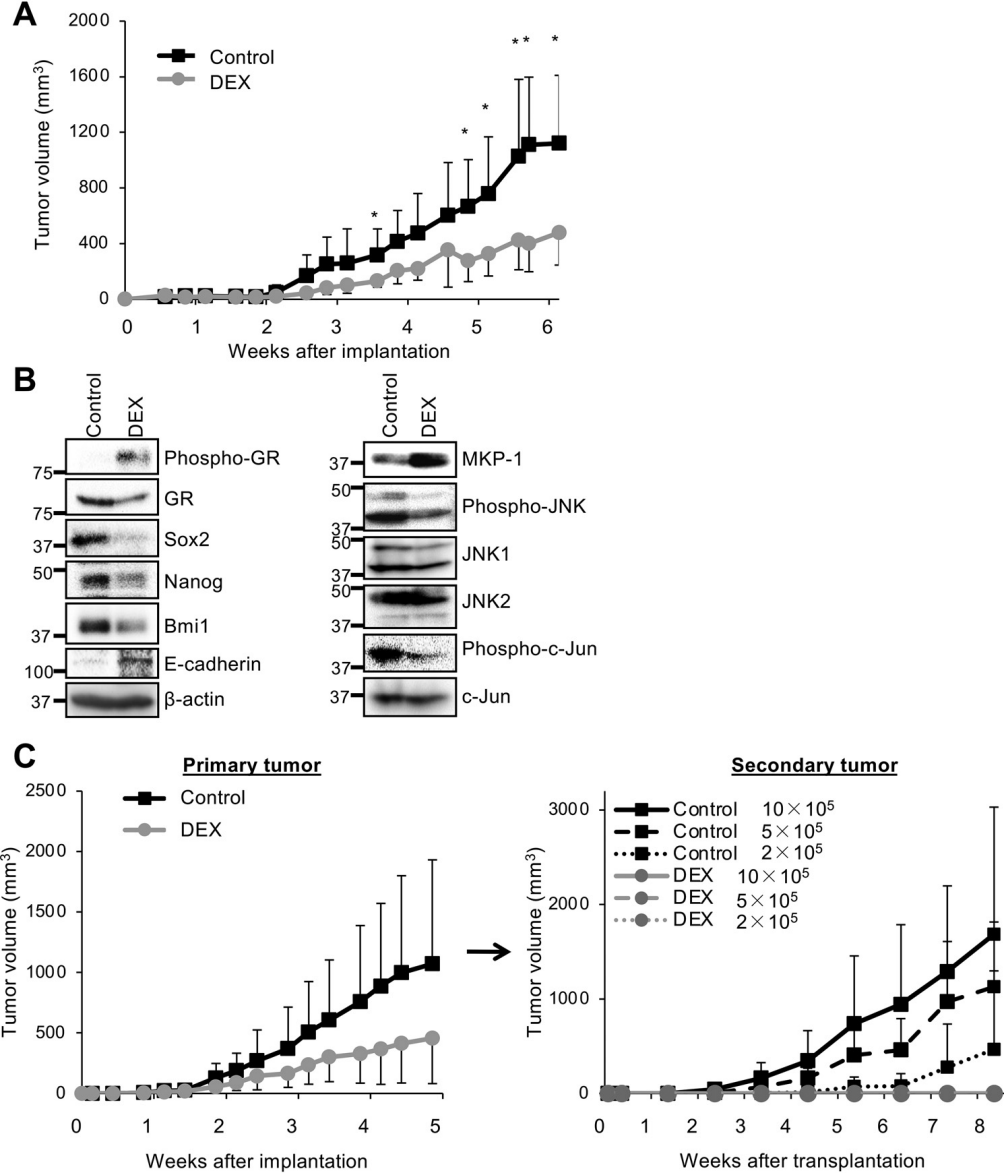
**Systemically administered dexamethasone inhibits JNK and eliminates CSCs in pancreatic tumor xenografts.***A*, mice implanted subcutaneously with 1 × 10^6^ viable PANC-1 CSLC cells (*n* = 5/group) were injected intraperitoneally with either vehicle only or 1 mg/kg DEX three times a week, starting the day after implantation. Subcutaneous tumor volumes were measured at the indicated time points. Data are shown as mean ± S.D. (*error bars*). *, *p* < 0.05. *B* and *C*, mice were treated as in *A* for 5 weeks (*n* = 6/group). Subcutaneous tumors (primary tumors) were excised and dissociated, and serial dilutions of dissociated tumor cells (2 × 10^5^, 5 × 10^5^, and 1 × 10^6^ viable cells) were transplanted subcutaneously into new mice (*n* = 5/group). *C*, volumes of primary tumors (*left*) and secondary tumors formed by transplantation of cells from primary tumors (*right*) were measured at the indicated time points. Data are shown as mean ± S.D. *B*, the indicated proteins were detected in dissociated primary tumor cells by immunoblotting.

Having demonstrated that the CSC-inhibitory effects of dexamethasone are mediated by the GR–MKP-1–JNK axis *in vitro*, we next investigated whether dexamethasone could inhibit CSCs *in vivo*. To this end, we tested the effect of systemic dexamethasone administered at a clinically relevant dose (1 mg/kg, three times a week) on tumor formation initiated by pancreatic CSCs. Systemic dexamethasone treatment was started following the implantation of pancreatic CSCs into recipient mice and significantly inhibited the growth of xenograft tumors compared with the vehicle-only control (Fig. 4*A*). Dexamethasone may therefore exert a CSC-inhibitory effect *in vivo*. Notably, whereas the above dose of dexamethasone was well-tolerated and did not affect the body weight of mice for at least 6 weeks (Fig. S6), the growth of dexamethasone-treated tumors appeared to have become blunted by 5 weeks (Fig. 4*A*), which suggests that, by this time point, dexamethasone had substantially reduced CSCs within the tumors that fuel tumor growth. To test this possibility, we performed an expression analysis on xenograft tumors from mice treated systemically for 5 weeks with the above dose of dexamethasone. We observed reduced expression of stem cell markers (Sox2, Nanog, and Bmi1) accompanied by increased expression of E-cadherin in dexamethasone-treated tumors (Fig. 4*B*), suggesting that dexamethasone reduced the CSC population within tumors by promoting differentiation. We also observed reduced levels of phosphorylated JNK and c-Jun along with increased levels of phosphorylated GR and MKP-1 in dexamethasone-treated tumors (Fig. 4*B*). Thus, dexamethasone most likely promotes differentiation of CSCs *in vivo* and *in vitro* by the same mechanism.

To determine whether dexamethasone eliminates CSCs within tumors *in vivo*, we conducted a secondary tumor formation assay to measure the frequency of CSCs within a tumor. In this assay, tumor cells from primary tumors are transplanted into secondary recipient animals after serial dilution (40). Whereas transplantation of as few as 2 × 10^5^ cells from primary tumors treated only with vehicle was sufficient to initiate a secondary tumor, indicating that the transplanted cells included CSCs capable of tumor initiation, transplantation of as many as 1 × 10^6^ cells from primary tumors treated as described above with dexamethasone failed to form any tumors (Fig. 4*C*). The data imply that the systemic dexamethasone dosing schedule caused a >5-fold reduction in the CSC population within primary tumors. Collectively, these findings suggest that the mechanism of dexamethasone's *in vitro* CSC-inhibitory effect may also be operating *in vivo* when dexamethasone is administered at clinically relevant doses.

### Dexamethasone sensitizes pancreatic cancer stem cells to chemotherapeutic agents by inhibiting survivin

**Figure 5:**
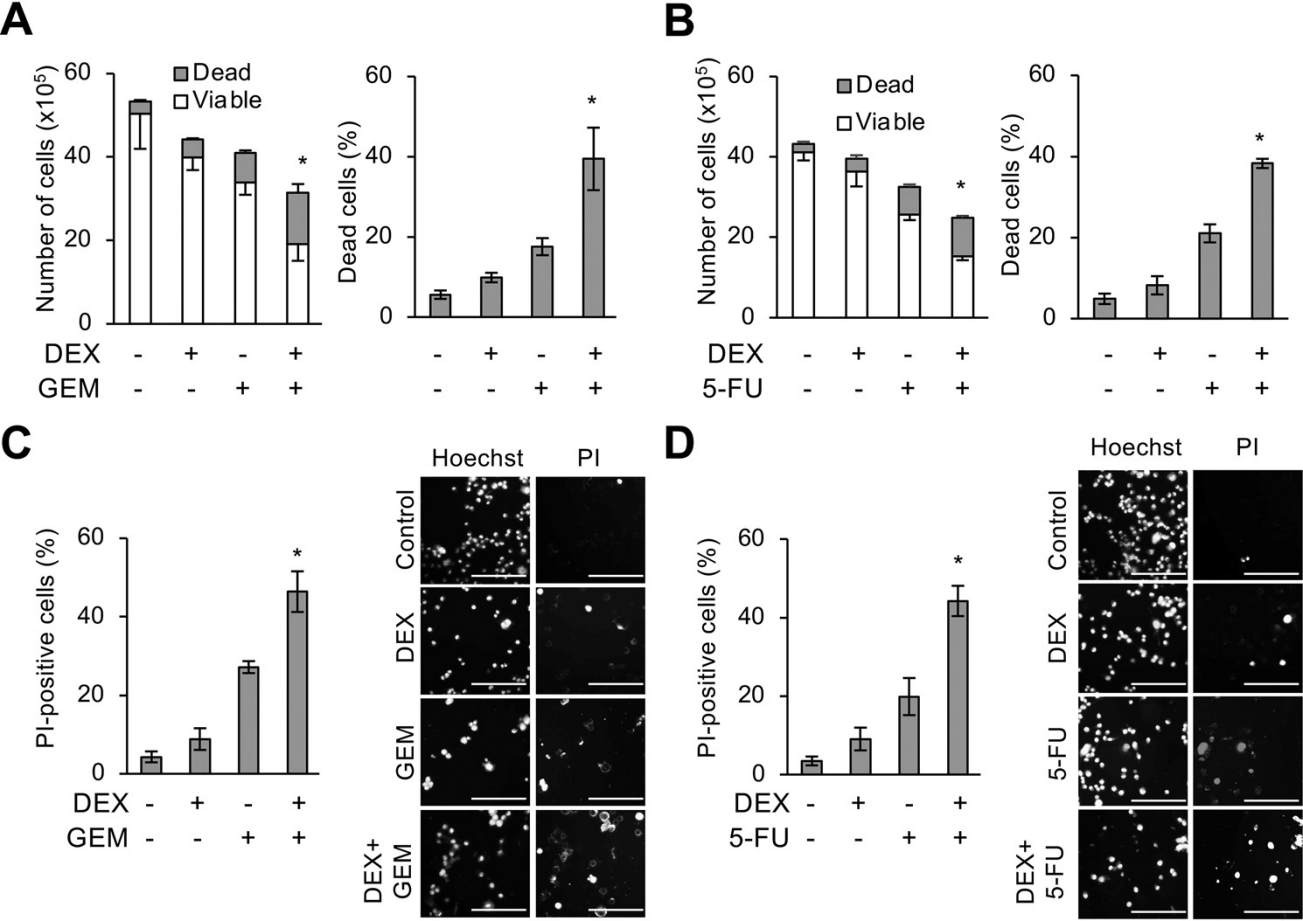
**Dexamethasone sensitizes pancreatic CSCs to gemcitabine and 5-fluorouracil.** PANC-1 CSLC cells were either pretreated with 1 μm DEX or left untreated for 6 days were subsequently treated with either 1 μm GEM or 10 μm 5-FU or left untreated as indicated for 3 days in the absence of DEX. *A* and *B*, viable and dead cell numbers (*left*) and percentage of dead cells (*right*) were determined by trypan blue staining. *C* and *D*, cell death assay using PI. *Left*, percentage of PI-positive (dead) cells relative to Hoechst-positive (both viable and dead) cells. *Right,* representative fluorescence images of Hoechst-positive (*left columns*) and PI-positive (*right columns*) cells. *Scale bars*, 200 μm. Data are shown in graphs as means ± S.D. (*error bars*) from triplicate samples of a representative experiment. *, *p* < 0.05.

**Figure 6:**
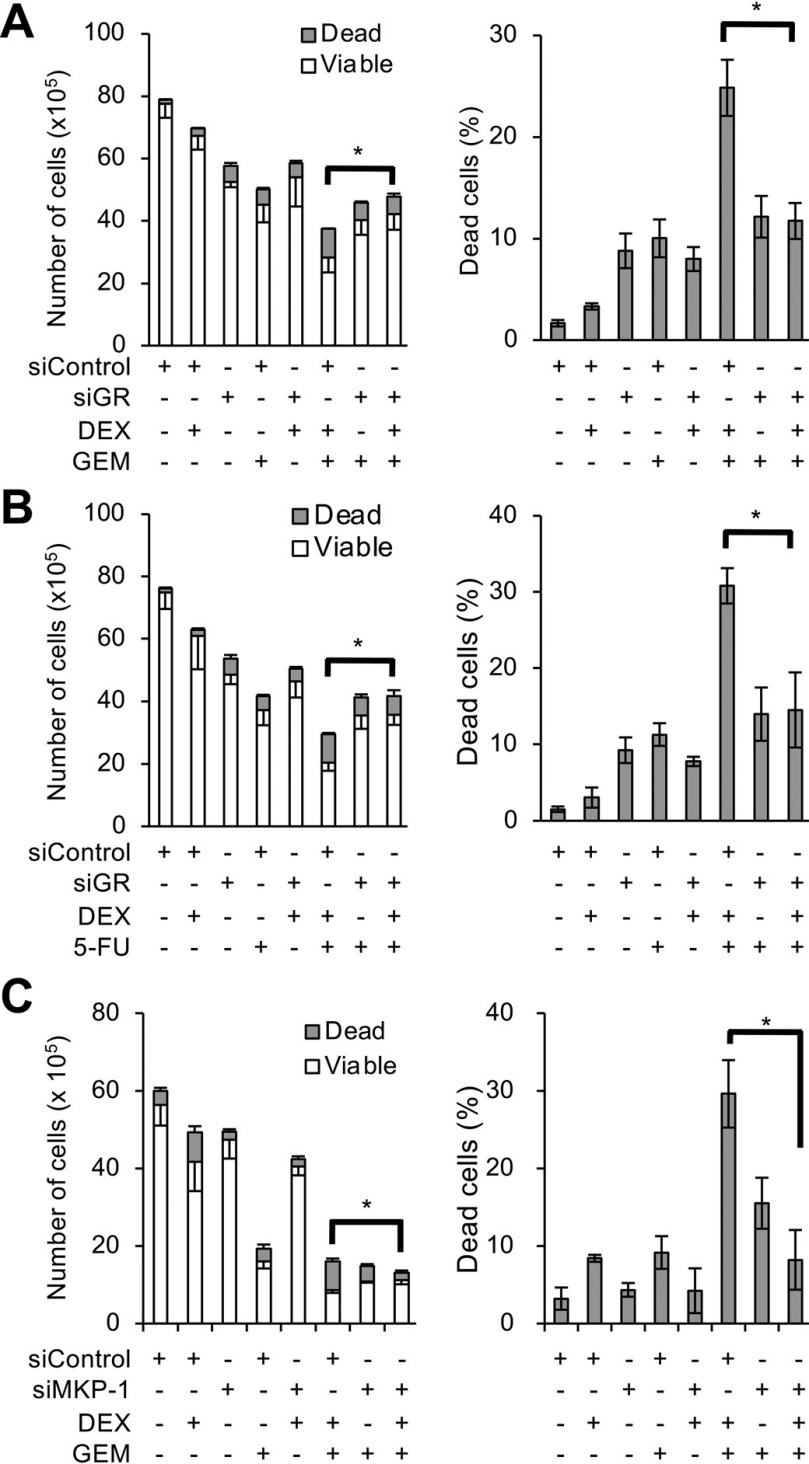
**Dexamethasone chemosensitizes pancreatic CSCs via glucocorticoid receptor and MKP-1.***A* and *B*, PANC-1 CSLC cells transiently transfected with either siRNA against the glucocorticoid receptor gene (*siGR*, #*2*) or control siRNA (*siControl*) for 24 h were either treated with 1 μm DEX or left untreated for 6 days. Cells were subsequently treated with either 1 μm GEM (*A*) or 10 μm 5-FU (*B*) or left untreated for 3 days. *C*, PANC-1 CSLC cells transiently transfected with either an siRNA against MKP-1 (*siMKP-1*, #*1*) or control siRNA (*siControl*) for 24 h were either treated with 1 μm DEX or left untreated for 6 days. Cells were subsequently either treated with 1 μm GEM or left untreated for 3 days. Cells treated as in *A–C* were stained with trypan blue to determine viable and dead cell numbers (*left*) and percentage of dead cells (*right*). Data are shown as means ± S.D. (*error bars*) from triplicate samples of a representative experiment. *, *p* < 0.05.

**Figure 7:**
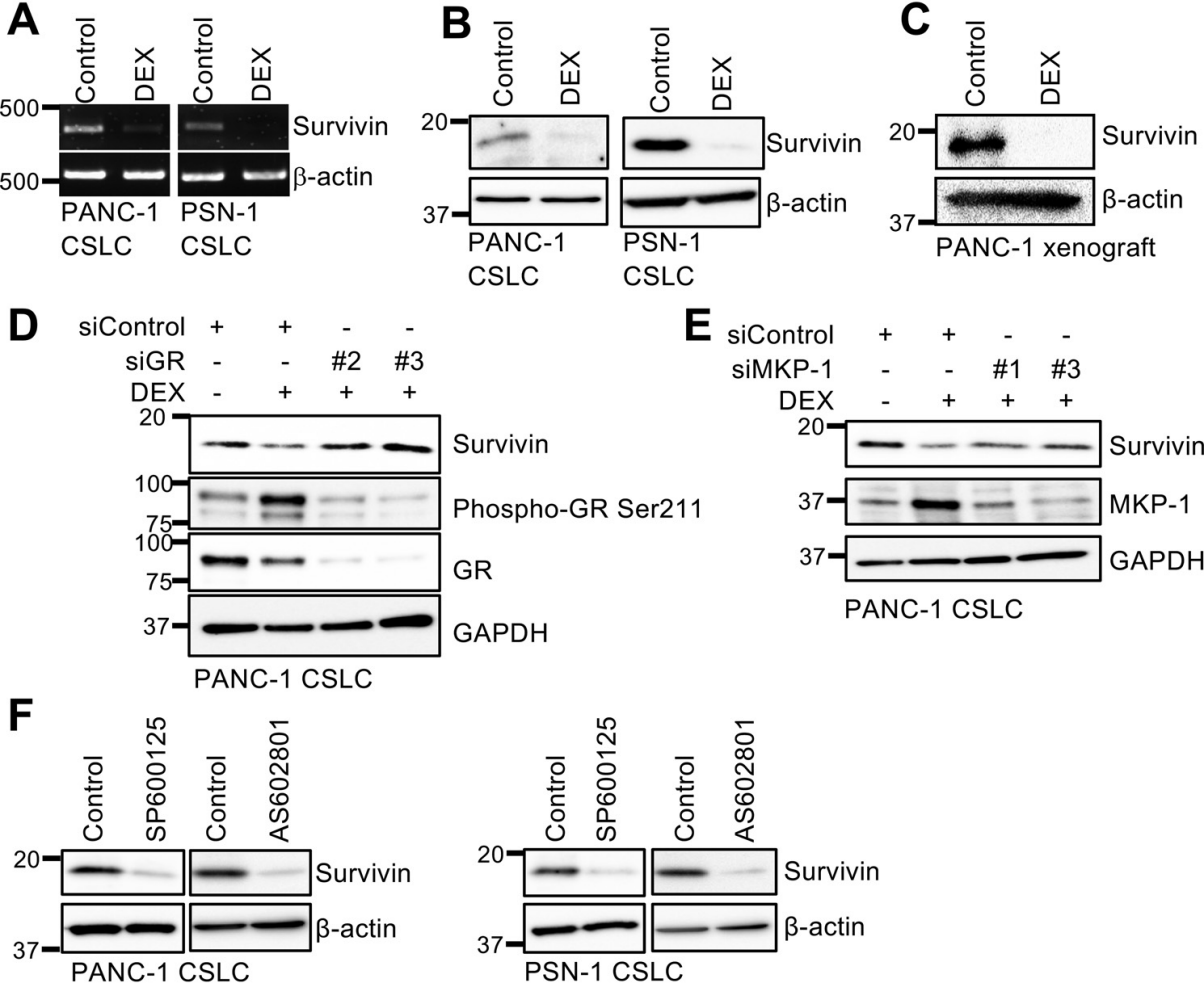
**Role of GR–MKP-1–JNK axis in dexamethasone-induced inhibition of survivin in pancreatic CSCs.***A* and *B*, survivin expression was detected in cells cultured either with 1 μm DEX or without DEX (*Control*) for 6 days by either RT-PCR (*A*) or immunoblotting (*B*). *C*, mice implanted subcutaneously with 1 × 10^6^ of viable PANC-1 CSLC cells were injected intraperitoneally with either vehicle only or 1 mg/kg DEX three times a week for 5 weeks starting the day after implantation. Subcutaneous tumors were excised and subjected to immunoblotting for survivin. *D* and *E*, PANC-1 CSLC cells transiently transfected with either siRNAs specific for GR (*siGR*, #*2* and #*3*) (*D*) or MKP-1 (*siMKP-1*, #*1* and #*3*) genes (*E*), or with control siRNA (*siControl*) for 24 h were either treated with 1 μm DEX or left untreated for 6 days before immunoblotting for the indicated proteins. *F*, survivin levels were determined by immunoblotting in cells cultured in the absence (*Control*) or presence of either SP600125 (20 μm) or AS602801 (7.5 μm) for 6 days.

CSCs have been closely associated with resistance to conventional cancer chemotherapy (3, 41, 42). Pretreatment with dexamethasone, which we have thus far shown to promote CSC differentiation, could therefore sensitize pancreatic CSCs to chemotherapeutic agents such as gemcitabine and 5-fluorouracil (5-FU), which are antimetabolites commonly used to treat patients with pancreatic cancer. When pancreatic CSCs pretreated with dexamethasone for 6 days were treated with either gemcitabine or 5-FU in the absence of dexamethasone, the inhibitory effects of these chemotherapeutic agents on the overall viability of the cells were accompanied by an increase in the proportion of dead cells, indicating that dexamethasone pretreatment sensitized pancreatic CSCs to the cytotoxic effects of gemcitabine and 5-FU (Fig. 5, *A–D*). Significantly, chemosensitization by dexamethasone was abrogated by knocking down GR (Fig. 6, *A* and *B*) or MKP-1 (Fig. 6*C*), suggesting its dependence on GR-mediated MKP-1 expression. Because our previous studies demonstrated a pivotal role for survivin, a member of the inhibitor of apoptosis (IAP) protein family, in the chemoresistance of pancreatic CSCs to gemcitabine and 5-FU (18, 20, 43), we determined whether survivin is involved in chemosensitization by dexamethasone. We could reproduce the results of our previous studies in the current study and confirmed that pancreatic CSCs express survivin. Moreover, knockdown of endogenous survivin expression was sufficient to sensitize them to gemcitabine and 5-FU (Fig. S7, A–E). Additionally, we found in this study that dexamethasone reduces endogenous survivin expression in pancreatic CSCs at the mRNA (Fig. 7*A*) and protein (Fig. 7*B*) levels, both *in vitro* and *in vivo* (Fig. 7*C*). Furthermore, we also noticed in this study increased survivin expression upon gemcitabine treatment, which was also inhibited by dexamethasone pretreatment similarly to the basal level of survivin expression (Fig. S8). We next determined whether the GR–MKP-1–JNK axis is involved in the regulation of survivin expression in pancreatic CSCs similarly to their stem cell state. The critical role of this axis in survivin expression was demonstrated by observations that knockdown of either GR or MKP-1 hampered dexamethasone-induced reduction in survivin expression (Fig. 7, *D* and *E*), whereas treatment with pharmacological JNK inhibitors without dexamethasone was sufficient to reduce survivin expression (Fig. 7*F*) in pancreatic CSCs. Unexpectedly, we found that activated JNK protein expression, which abolished dexamethasone's inhibitory effects on the stemness of pancreatic CSCs (Fig. 3*E*), failed to block the dexamethasone-induced reduction in survivin expression (Fig. S9). Because activated JNK is derived from JNK1, we speculated that JNK1 may not be involved in regulating survivin expression. Consistent with this idea, the results of knockdown experiments demonstrated that survivin expression is primarily JNK2-dependent in contrast to Sox2 and E-cadherin expression, which are mainly JNK1-dependent (Fig. S10). Thus, dexamethasone most likely reduces survivin expression in pancreatic CSCs via GR– and MKP-1–mediated inhibition of JNK2.

### Systemic treatment with both dexamethasone and gemcitabine suppresses development and growth of tumors from pancreatic CSCs

**Figure 8:**
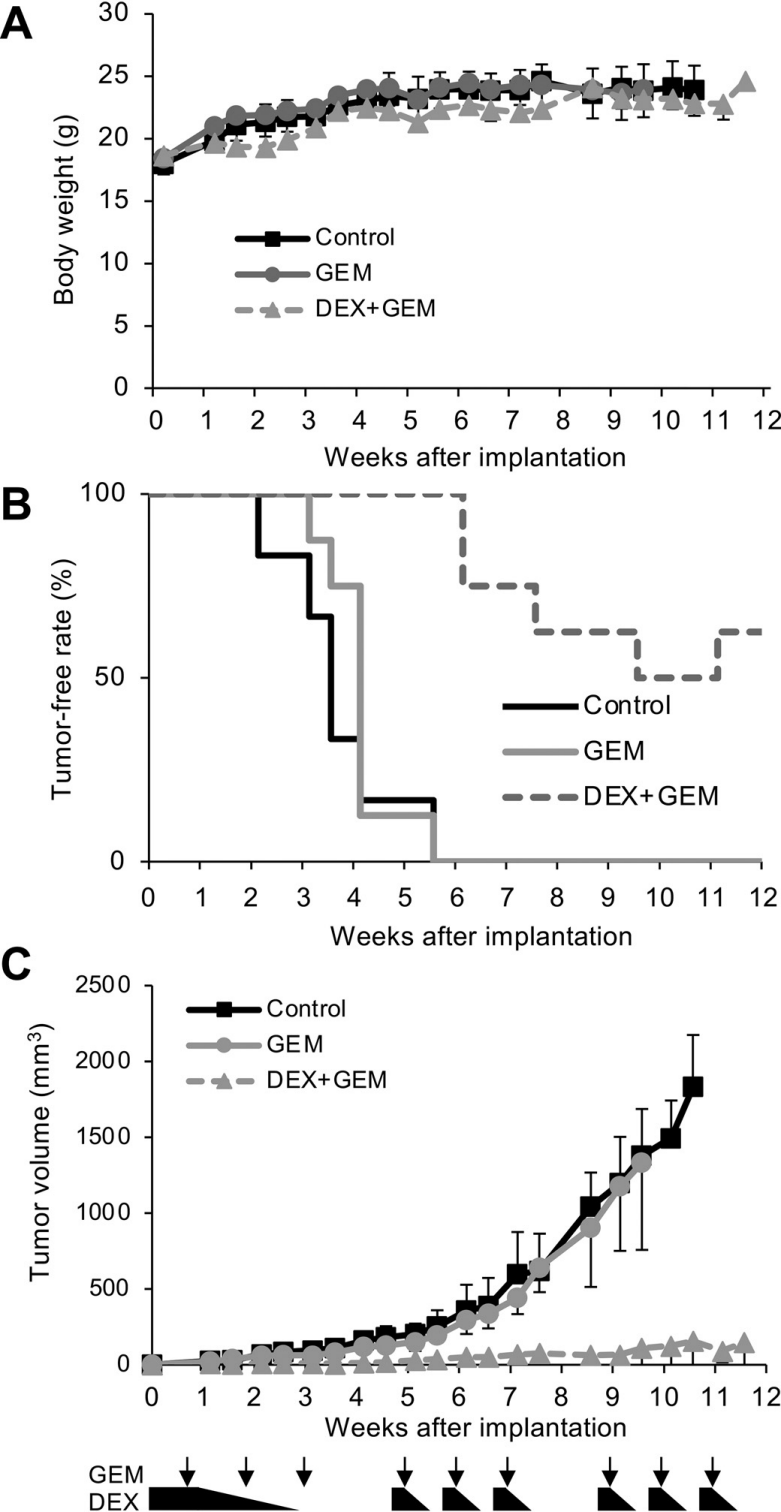
**Dexamethasone and gemcitabine together effectively eliminate pancreatic CSCs *in vivo*.***A–C*, mice implanted subcutaneously with 1 × 10^6^ viable PANC-1 CSLC cells (*n* = 6 for the control group, 8 each for the DEX and DEX + GEM groups) were either treated with GEM alone, treated with a combination of GEM + DEX, or left untreated (*Control*) as depicted schematically (see Fig. S10 for treatment protocol details). Body weight (*A*) and tumor volumes (*C*) at the indicated time points are shown. Data are shown as mean ± S.D. (*error bars*). The tumor-free rate is presented in *B*.

The data we have presented so far suggest that the combination of dexamethasone with antimetabolites such as gemcitabine and 5-FU is a rational approach to eliminating CSCs within tumors not only functionally by rendering them non-CSCs but also physically by inducing cell death. We therefore tested the efficacy as well as the safety of this combination in a clinically oriented setting. Because adjuvant gemcitabine monotherapy is effective for treating patients with macroscopic complete removal of pancreatic cancer but nevertheless results in a high rate of disease recurrence (44), we wished to determine whether systemic dexamethasone concomitant with gemcitabine can improve the treatment outcome by inhibiting tumor growth from CSCs. To this end, nude mice implanted with pancreatic CSCs were treated with cycles of gemcitabine three times a week either with or without concomitant systemic dexamethasone. Gemcitabine was used at 40 mg/kg, three times a week, which is roughly one-third of the maximum dose, when translated to a human equivalent, used in the treatment of pancreatic cancer (44). Dexamethasone was administered initially at 1 mg/kg/day for the first 9 days before tapering over a 12-day period (a total of 11.95 mg/kg over 3 weeks) (45), followed by weekly cycles of dexamethasone (1 mg/kg for 2 days tapered over a 3-day period = 2.875 mg/kg/week), which was almost the same dose as what was used earlier in this study (1 mg/kg, 3 times a week = 3 mg/kg/week) (Figure 4, Figure 5, Figure 6, Figure 7*C*). Not only was the combination of dexamethasone and gemcitabine at these doses tolerated by the mice, as demonstrated by the lack of significant weight change (Fig. 8*A*), but strikingly, we found that the combination dramatically inhibited the development and growth of tumors in sharp contrast to gemcitabine monotherapy, which showed virtually no inhibitory effect (Fig. 8 (*B* and *C*) and Fig. S11). Whereas by 4 weeks post-implantation, implantation of CSCs invariably led to the development of tumors that continued to grow progressively when recipient mice were either treated with gemcitabine monotherapy or left untreated, five of eight implantation sites were tumor-free even after 11 weeks when the mice were treated with the combination of dexamethasone and gemcitabine (Fig. 8*B* and Fig. S11). Moreover, the tumors that developed in the combination treatment group were much smaller than those that developed in the other two groups and did not show progressive growth (Fig. 8*C* and Fig. S11). These results clearly indicate that systemic dexamethasone concomitant with gemcitabine effectively inhibits the development and growth of tumors from pancreatic CSCs without causing adverse effects in nude mice.

## Discussion

Glucocorticoids, including dexamethasone, have been widely and commonly used to treat nonhematologic malignancies to prevent and manage cancer- or treatment-related symptoms in patients with advanced cancer (21, 23). However, despite clinical studies aiming to determine the effect of glucocorticoids on solid tumor growth or response *per se* in a variety of nonhematologic cancers, their efficacy has not yet been proven except for the modest benefit of glucocorticoid monotherapy in breast and prostate cancer treatment (21, 23). However, importantly, those clinical studies were conducted mostly on patients with advanced cancer, for whom the growth of the remaining tumor bulk as a result of limited rounds of proliferation of non-CSCs, rather than *de novo* tumor development fueled by infinitely self-renewing CSCs, is thought to limit their survival. Thus, it remains unknown whether glucocorticoids have clinically significant anti-CSC activities. They could therefore prove effective in preclinical and clinical studies properly designed to evaluate their anti-CSC potential.

In this study, we addressed this question and demonstrated that dexamethasone inhibits cellular properties associated with the self-renewal capacity of CSCs derived from pancreatic, lung, and ovarian cancers and promotes their differentiation *in vitro*. Furthermore, using pancreatic CSCs as a model, we found that inhibition of the JNK pathway as a result of GR-dependent transcriptional activation of the MAPK phosphatase MKP-1 is a primary mechanism involved in the CSC-inhibitory effect of dexamethasone, although our results do not exclude the contribution of other MKP-1 substrates, including ERK and p38 MAPK. Whereas JNK has been shown to play a pivotal role in CSC maintenance in various human cancers (28, 29, 30, 31, 32, 33, 34), the role of MKP-1 in CSCs, in contrast, remains mostly unclear, except that two recent studies reported that high MKP-1 expression is associated with the loss of stemness in glioma stem cells (46, 47). Hence, the results of this study together with these studies may underscore the role of MKP-1 as a negative regulator of CSCs that inhibits JNK. We also demonstrated that systemic dexamethasone reduces the tumor-initiating population within primary xenograft tumors established from pancreatic CSCs, suggesting that dexamethasone eliminates pancreatic CSCs both *in vitro* and *in vivo*. Consistent with our findings, a previous study showed that systemic dexamethasone inhibits the development of pancreatic intraepithelial neoplasias (PanINs) in a genetically engineered mouse model of pancreatic cancer (48). Although inhibition of inflammation was proposed as the mechanism for dexamethasone's effect on the development of PanINs (48), our findings suggest that elimination of pancreatic CSCs through JNK pathway inhibition is a plausible mechanism, although these two mechanisms are not mutually exclusive when the JNK pathway's pivotal role in inflammation is considered. Given the well-documented resistance of CSCs to conventional chemotherapy, we also investigated whether and how dexamethasone affects the sensitivity of pancreatic CSCs to gemcitabine and 5-FU, key chemotherapeutic agents used to treat pancreatic cancer, to determine whether there is any significant interaction between dexamethasone and these drugs. Because earlier studies suggested that dexamethasone may antagonize the cytotoxic effect of gemcitabine when cells are treated simultaneously with these drugs (49, 50), we examined in this study the effect of dexamethasone “pre-”treatment on the growth-inhibitory effects of chemotherapeutic agents. Significantly, our results demonstrated that dexamethasone pretreatment for as long as 6 days was sufficient to chemosensitize pancreatic CSCs to gemcitabine and 5-FU most likely through the inhibition of survivin expression, a potent apoptotic inhibitor implicated in chemoresistance (51). Our results may thus imply that the modulatory effects of dexamethasone on the chemo-sensitivity/resistance of cancer cells could be dependent on the duration and timing of treatment. On the other hand, because the cell type–specific pro-apoptotic effects of glucocorticoids have been in part explained by the differential expression of GR isoforms that are capable of mediating the inhibition of the NF-κB–survivin axis by glucocorticoids (52), it may be interesting to investigate the possible contribution of NF-κB, a key player in inflammation, to pancreatic CSC chemoresistance.

Most importantly, we have successfully demonstrated that dexamethasone can have a therapeutic effect in a mouse pancreatic cancer xenograft model. Using a preclinical model simulating postoperative tumor recurrence whereby implanted pancreatic CSCs develop from a microscopic cluster of cells into a full-blown tumor, we tested gemcitabine monotherapy with and without concomitant administration of systemic dexamethasone. In contrast to the apparent ineffectiveness of gemcitabine when administered alone, gemcitabine in combination with dexamethasone enabled sustained inhibition of tumor development from CSCs, underscoring the inhibition of CSCs by dexamethasone. Gemcitabine's contribution remains unclear because dexamethasone monotherapy was not in-cluded in our study due to the lack of clinical feasibility. However, we surmise that gemcitabine helped eliminate CSCs that were incompletely differentiated yet rendered sensitive to it by dexamethasone as demonstrated *in vitro*, which might otherwise have reverted to CSCs due to plasticity (9). It should also be noted that the maximum daily dose of dexamethasone used in the preclinical model was well below the maximum daily dose approved by the United States Food and Drug Administration when converted to a human equivalent (53) and well-tolerated by mice even when administered concomitantly with the modest dose of gemcitabine used in this study. In addition, by starting dexamethasone before the formation of overt tumors, we could avoid impaired drug delivery associated with the formation of desmoplastic stroma that progresses with tumor growth (54, 55). Put together, the results of the preclinical animal study strongly support the idea that the therapeutic effect of dexamethasone would be best appreciated in patients with pancreatic cancer treated with a gemcitabine (or 5-FU)–based regimen after tumor resection. The survival of such patients is most likely to be affected by microscopic, residual CSCs.

Strikingly, while our study was in progress, a significant association between intraoperative dexamethasone administration and long-term survival in patients with pancreatic cancer undergoing surgical resection was demonstrated in two independent studies (56, 57). Although both studies are retrospective in nature, their results agree with those of our preclinical study and thus provide clinical evidence that supports our idea that dexamethasone benefits patients with resected pancreatic cancer. Meanwhile, our *in vitro* data show that more than several days of dexamethasone exposure was required to cause the entire pancreatic CSC population to commit to differentiation; thus, it would be surprising if a single intraoperative administration of dexamethasone alone is sufficient to improve survival. In this regard, other factors associated with perioperative management may promote dexamethasone's inhibitory effects on CSCs. Consistent with this idea, the perioperative use of dexamethasone in combination with flurbiprofen axetil, a nonsteroidal anti-inflammatory drug, has been associated with improved long-term survival in patients undergoing surgery for non-small-cell lung cancer (58).

Glucocorticoids reportedly promote metastasis in preclinical models of metastatic breast cancer through GR-mediated activation of ROR1 specifically in metastatic breast cancer cells (59). However, in clinical settings, glucocorticoid monotherapy has been associated with clinical benefits in breast cancer, and there is no clinical evidence associating glucocorticoid use with poorer survival in patients with breast cancer (21, 23). Furthermore, we did not observe ROR1 induction by dexamethasone in the pancreatic, lung, and ovarian CSCs used in our study (not shown). Our data, taken together with relevant clinical data, suggest the potential therapeutic benefit of dexamethasone as a CSC-targeting agent for treating pancreatic cancer and, possibly, some other human cancers.

Another important implication of our study is the potential role of JNK in inflammation-associated carcinogenesis. A large body of evidence indicates that chronic inflammatory processes are involved in the development of a variety of human cancers, including pancreatic cancer, and play decisive roles in virtually all stages of tumor development (60, 61). However, much remains unknown regarding the mechanisms by which inflammation promotes tumor development. Inflammation may enhance tumor initiation by expanding the CSC population, yet the molecular pathways linking inflammation to tumor initiation are poorly understood except for the suggested involvement of a handful of molecules, including STAT3, NF-κB, and Wnt/β-catenin (61, 62). In this study, we have shown that dexamethasone, a representative anti-inflammatory drug, inhibits tumor development by pancreatic CSCs by affecting the MKP-1–JNK axis which is known to play a central role in mediating the anti-inflammatory actions of glucocorticoids (63). Our findings thus suggest that the JNK pathway contributes to inflammation-induced carcinogenesis as a key modulator of the CSC population, although we have not yet tested this hypothesis directly using an experimental inflammation-induced cancer model. Nevertheless, there is further circumstantial evidence that implicates the JNK pathway in CSC maintenance during tumor development associated with inflammation. Similarly to pancreatic cancer, inflammation also plays a role in the development of hepatocellular carcinoma (HCC) (64, 65). HCC provides a compelling example of JNK pathway involvement in the initiation and progression of cancer (66). Moreover, a growing body of evidence now suggests a critical role for JNK in maintaining the CSC population in HCC (32, 34, 67). Apparently, all of these findings can be explained if we assume that inflammation-induced JNK activation promotes tumorigenesis by expanding the CSC pool. Focusing on the JNK pathway's role in inflammation-induced carcinogenesis may therefore elucidate the underlying mechanism and lead to rational therapies for inflammation-associated human cancers.

In conclusion, we have demonstrated that dexamethasone, through GR– and MKP-1–mediated inhibition of JNK, drives pancreatic CSC differentiation into non-CSCs and sensitizes CSCs to chemotherapeutic agents by inhibiting survivin. Our data also show that systemic dexamethasone in combination with gemcitabine exerts a remarkable therapeutic effect in a preclinical pancreatic cancer model under treatment conditions properly designed to evaluate the anti-CSC effect of test drugs. Our findings have uncovered a hitherto unrecognized potential of dexamethasone as a CSC-targeting drug and suggest that dexamethasone could be a viable therapeutic solution to prevent tumor recurrence from CSCs, warranting future evaluation of dexamethasone's anti-CSC effects in well-designed clinical trials. Our results also indicate that JNK-dependent maintenance of CSCs may link inflammation and tumor initiation and thus provide novel insights into the mechanisms underlying inflammation-induced cancer.

## Experimental procedures

### Antibodies and reagents

Antibodies against Sox2 (#3579), Nanog (#4903), Bmi1 (#6964), E-cadherin (#3195, for immunocytochemistry), glucocorticoid receptor (#12041), phospho-glucocorticoid receptor (Ser-211) (#4161), c-Jun (#9165), phospho-c-Jun (Ser-63) (#9261), phospho-SAPK/JNK (Thr-183/Tyr-185) (#4668), survivin (#2808), GFAP (#3670), phospho-Smad2 (Ser-465/467) (#3108), Smad2/3 (#8685), and GAPDH (#5174) were purchased from Cell Signaling Technology, Inc. (Beverly, MA, USA). Antibodies against JNK1 (sc-474), JNK2 (sc-7345), E-cadherin (sc-8426, for immunoblotting), and MKP-1 (sc-370) were from Santa Cruz Biotechnology, Inc. (Dallas, TX, USA). The antibody against nestin (MAB5326) was purchased from Merck Millipore (Darmstadt, Germany). Anti-CD133 (W6B3C1) was purchased from Miltenyi Biotech (Bergisch Gladbach, Germany). Anti-β-actin (A1978) was from Sigma–Aldrich. Dexamethasone (DEX) was purchased from Fuji Pharma Co., Ltd. (Tokyo, Japan). Prednisolone (PSL), 5-FU, and gemcitabine (GEM) were from Sigma–Aldrich. DEX, PSL, 5-FU, and GEM were dissolved in PBS (for DEX and PSL) or DMSO (for 5-FU and GEM) to prepare 1, 10, 200, and 1 mm stock solutions for *in vitro* use, respectively.

### Cell culture

The human pancreatic cancer cell line PANC-1 was obtained from the Cell Resource Center for Biomedical Research, Institute of Development, Aging, and Cancer, Tohoku University. PSN-1 was a kind gift from Dr. T. Yoshida at the National Cancer Center Research Institute, who originally established the cell line from pancreatic adenocarcinoma tissue (68). The human non-small-cell lung cancer cell line A549 was obtained from the Riken BioResource Center (Tsukuba, Japan). The human ovarian cancer cell line A2780 was a kind gift from Dr. T. Tsuruo (Institute of Molecular and Cellular Biosciences, University of Tokyo, Japan) and Drs. R. F. Ozols and T. C. Hamilton (National Institutes of Health) (69). These cell lines were maintained in DMEM/F-12 medium supplemented with 10% fetal bovine serum (FBS; Sigma) (28, 70, 71). Normal human IMR90 fetal lung fibroblasts were obtained from the American Type Culture Collection (ATCC, Manassas, VA, USA) and maintained in DMEM supplemented with 10% FBS. Furthermore, the culture medium was supplemented with 100 units/ml penicillin and 100 μg/ml streptomycin. The establishment of human cancer stem cells used in this study (PANC-1 CSLC, PSN-1 CSLC, A549 CSLC, and A2780 CSLC) was reported previously (17, 28, 70, 71). Rat cortical neural stem cells were purchased from R&D Systems (Minneapolis, MN, USA). These cells were maintained under monolayer stem cell culture conditions, as reported previously (17, 28, 70, 71). Briefly, cells were cultured on collagen-I–coated dishes (IWAKI, Tokyo, Japan) in the stem cell culture medium (DMEM/F-12 medium supplemented with 1% B27 (Thermo Fisher Scientific), 20 ng/ml EGF and FGF2 (Peprotech Inc., Rocky Hill, NJ, USA), d-(+)-glucose (final concentration, 26.2 mm), l-glutamine (final concentration, 4.5 mm), 100 units/ml penicillin, and 100 μg/ml streptomycin), except that rat cortical neural stem cells were cultured on dishes coated with Geltrex® (Thermo Fisher Scientific). Stem cell culture medium was changed approximately every 3 days, and EGF and FGF2 were added to the culture medium every day. *In vitro* drug treatment was performed using cells in monolayer culture. The authenticity of PANC-1 CSLC, PSN-1 CSLC, A549 CSLC, and A2780 CSLC was confirmed by the genotyping of short tandem repeat (STR) loci (Bio-Synthesis Inc., Lewisville, TX, USA), followed by comparison with the ATCC STR database (RRID:SCR_019203) for human cell lines. All IMR90 and rat neural cortical stem cell experiments were performed using cells with a low passage number (<8).

### Flow cytometric analysis

Flow cytometric analysis was conducted as described previously (28, 72, 73). For analyses of CD133 expression, dissociated cells were washed with ice-cold PBS, fixed with 4% (w/v) paraformaldehyde for 10 min at room temperature (RT), and washed again with PBS. The cells were blocked in FCM buffer (0.5% (w/v) BSA and 0.1% (w/v) NaN_3_ in PBS) for 1 h, followed by three PBS rinses and further incubation with anti-CD133 antibody in the FCM buffer overnight at 4 °C and then incubation with Alexa Fluor® 488 goat anti-mouse IgG for another 1 h at RT. Cells exhibiting signal for CD133 above the gate established by the isotype control were considered CD133-positive. At least 1 × 10^4^ cells were evaluated and gated using side and forward scatters to identify viable cell populations. All flow cytometry analysis experiments were run on the FACSCanto^TM^ II flow cytometer (BD Biosciences), and the data were analyzed using FlowJo software, version 7.6.5 (FlowJo LLC., Ashland, OR, USA).

### Immunoblot analysis

Immunoblot analysis was conducted as described previously (15, 28, 72, 73). Cells (2–5 × 10^5^) and tumor tissue were washed with ice-cold PBS and lysed in RIPA buffer (10 mm Tris-HCl (pH 7.4), 0.1% SDS, 0.1% sodium deoxycholate, 1% Nonidet P-40, 150 mm NaCl, 1 mm EDTA, 1.5 mm Na_3_VO_4_, 10 mm NaF, 10 mm sodium pyrophosphate, 10 mm sodium β-glycerophosphate, and 1% protease inhibitor mixture set III (Sigma)). The same volume of 2× Laemmli buffer (125 mm Tris-HCl (pH 6.8), 4% SDS, 10% glycerol, and 10% 2-mercaptoethanol) was immediately added and boiled at 95 °C for 10 min. The tumors were carefully dissected, minced, and homogenized in a Potter–Elvehjem homogenizer with a BioMasher® II (Nippi, Tokyo, Japan). After centrifugation for 10 min at 14,000 × *g* at 4 °C, the supernatants were recovered as cell lysates. The protein concentrations of the cell lysates were measured using a BCA protein assay kit (Thermo Fisher Scientific). Cell lysates containing equal amounts of protein were separated by SDS-PAGE and transferred to a polyvinylidene difluoride membrane. The membrane was probed with a primary antibody and then with an appropriate HRP-conjugated secondary antibody according to the protocol recommended by the manufacturer of each antibody. Immunoreactive bands were visualized using Immobilon Western Chemiluminescent HRP Substrate (Millipore) and detected by a ChemiDoc Touch (Bio-Rad).

### Immunofluorescence analysis

The protocol of immunofluorescence analysis was modified from previous reports (17, 71, 74). Briefly, cells (0.2–0.5 × 10^5^) were seeded onto Geltrex-coated coverslips in 12-well dishes and used for experiments. After cells were fixed with 4% (w/v) paraformaldehyde for 10 min at RT and washed with PBS, they were permeabilized and blocked with 0.4% Triton X-100, 2% FBS in PBS for 15 min at RT. After washing with PBS three times, the cells were incubated with a primary antibody in PBS containing 2% FBS at 4 °C overnight and then incubated with secondary antibodies in the same buffer for 1 h at RT. Fluorescence images were acquired using a confocal laser-scanning microscope (FLUOVIEW FV10i, OLYMPUS, Tokyo, Japan).

### Sphere formation assay

The sphere formation assay was performed as described previously (15, 28, 72). After cells treated with or without dexamethasone in monolayer culture were dissociated, single cells were serially diluted in the stem cell culture medium and seeded into ultra-low-attachment coated 96-well plates (Corning Inc., Corning, NY, USA) such that each well contained a single cell. Wells containing a single cell were marked under a phase-contrast microscope the next day, and 1 week after seeding, the percentage of marked wells with a sphere relative to the total number of marked wells was calculated.

### Transfection of siRNA or plasmids

siRNA against human GRα (NR3C1; #2 HSS178979, #3 HSS178980), MKP-1 (DUSP1; #1 HSS102982, #2 HSS102983, #3 HSS102984), survivin (BIRC5; HSS179403), and medium GC duplex #2 of Stealth RNAi^TM^ siRNA negative control duplexes (as a nontargeting control for siRNA experiments) were purchased from Thermo Fisher Scientific. Transfection of siRNAs was performed using Lipofectamine RNAiMAX (Thermo Fisher Scientific) according to the manufacturer's instructions. pcDNA3-FLAG-MKK7B2JNK1a1, which expresses an activated JNK1 protein, was a gift from Roger Davis (Addgene plasmid #19726) (75). To achieve sustained knockdown of the target genes or prolonged gene expression from the plasmid, siRNA/plasmid transfection was repeated 4 days after the initial transfection.

### RT-PCR analysis

RT-PCR analysis was performed as described previously (28). Total RNA was extracted using TRIzol (Thermo Fisher Scientific). Total RNA was reverse-transcribed into cDNA using the PrimeScript^TM^ first-strand cDNA synthesis kit (Takara Bio Inc., Shiga, Japan) according to the manufacturer's instructions. Amplification was performed by 28 cycles of 97 °C for 30 s, 58 °C for 30 s, and 72 °C for 20 s in a thermal cycler (Takara PCR Thermal Cycler Dice). RT-PCR analysis was performed using the following primers: MKP-1 (forward, 5′-GGATACGAAGCGTTTTCGGC; reverse, 5′-GGTTGTCCTCCACAGGGATG), survivin (forward, 5′-CCTTTCTCAAGGA-CCACCGCATCT; reverse, 5′-CGCACTTTCTCCGCAGTT-TCCT), and β-actin (forward, 5′-CCCATGCCATCCTGC-GTCTG; reverse, 5′-CGTCATACTCCTGCTTGCTG).

### Cell death assay

Viable and dead cells were identified by their ability and inability to exclude vital dyes, respectively (15, 73). Briefly, cells (1 × 10^5^) were treated with drugs, as described in the figure legends and stained with 0.2% trypan blue for 1 min at RT, and the numbers of viable and dead cells were counted using a hemocytometer. The percentage of dead cells was defined as 100 × (number of dead cells/(number of viable + dead cells)), and cell viability (%) was defined as 100 × (number of viable cells/(number of viable + dead cells)). Alternatively, cells were incubated *in situ* with propidium iodide (PI; 1 μg/ml) and Hoechst 33342 (10 μg/ml) for 10 min at 37 °C in a CO_2_ incubator to stain the dead cells and nuclei, respectively. Subsequently, the numbers of PI- and Hoechst-positive cells were scored under a fluorescence microscope (CKX41, Olympus, Tokyo, Japan), and the percentage of PI-positive (dead) cells relative to Hoechst-positive (both viable and dead) cells was calculated.

### Mouse studies

For subcutaneous implantation, 6–9-week-old male BALB/cAJcl-nu/nu mice (CLEA Japan Inc., Tokyo, Japan) were implanted subcutaneously in the flank region with cells suspended in 200 μl of sterilized PBS under avertin (0.375 g/kg intraperitoneally) anesthesia. After implantation, the recipient mice were monitored for general health status and presence of subcutaneous tumors. For serial transplantation, primary tumors treated as described in the figure legend were excised and, after being washed in chilled sterile PBS, were transferred into DMEM/F-12, minced with scissors, and incubated in Accutase (Sigma–Aldrich) for 30 min at 37 °C. After rinsing with DMEM/F-12, the cells were resuspended in DMEM/F-12 and filtered through a 70-μm strainer. The single-cell suspension was subcutaneously injected after cell number and viability were assessed. Alternatively, the cells were subjected to immunoblot analysis. For systemic administration, DEX and GEM were dissolved in PBS to prepare 0.2 and 16 mg/ml stock solutions, respectively. These stock solutions were diluted in PBS to prepare 200-μl solutions for each injection. The DEX or GEM solutions were injected intraperitoneally into nude mice. The tumor volume was determined by measuring tumor diameters (measurement of two perpendicular axes of tumors) using a caliper and calculated as ½ × (larger diameter) × (smaller diameter)^2^. As the subcutaneous injection of cell suspension may lead to the transient formation of a noncancerous lump of tissue that regresses spontaneously, in this study, animals were judged to have a tumor when the volume of the subcutaneous mass exceeded 100 mm^3^ (76, 77). All animal experiments were performed following a protocol approved by the Animal Research Committee of Yamagata University.

### Statistical analysis

Results are expressed as means and S.D., and data were analyzed using a Student's *t* test for comparisons between two groups and a one-way analysis of variance followed by Dunnett's test for comparisons of more than two groups. *p* values of <0.05 were considered significant and are indicated with *asterisks* in the figures.

## Data availability

All of the data are contained within the article.

the Ministry of Education, Culture, Sports, Science and Technology of Japan (18H02908) to Shuhei Suzuki, Masashi Okada, and Chifumi Kitanaka
the Ministry of Education, Culture, Sports, Science and Technology of Japan (20K07631) to Shuhei Suzuki, Masashi Okada, and Chifumi Kitanaka
the Ministry of Education, Culture, Sports, Science and Technology of Japan (17K15015) to Shuhei Suzuki, Masashi Okada, and Chifumi Kitanaka

## References

[1] Bray F., Ferlay J., Soerjomataram I., Siegel R.L., Torre L.A., Jemal A. (2018). Global cancer statistics 2018: GLOBOCAN estimates of incidence and mortality worldwide for 36 cancers in 185 countries. CA Cancer J. Clin.

[2] Dawood S., Austin L., Cristofanilli M. (2014). Cancer stem cells: implications for cancer therapy. Oncology (Williston Park).

[3] Shibata M., Hoque M.O. (2019). Targeting cancer stem cells: a strategy for effective eradication of cancer. Cancers (Basel).

[4] Garber K. (2018). Cancer stem cell pipeline flounders. Nat. Rev. Drug Discov.

[5] Waring M.J., Arrowsmith J., Leach A.R., Leeson P.D., Mandrell S., Owen R.M., Pairaudeau G., Pennie W.D., Pickett S.D., Wang J., Wallace O., Weir A. (2015). An analysis of the attrition of drug candidates from four major pharmaceutical companies. Nat. Rev. Drug Discov.

[6] Siegel R.L., Miller K.D., Jemal A. (2019). Cancer statistics, 2019. CA Cancer J. Clin.

[7] Rahib L., Smith B.D., Aizenberg R., Rosenzweig A.B., Fleshman J.M., Matrisian L.M. (2014). Projecting cancer incidence and deaths to 2030: the unexpected burden of thyroid, liver, and pancreas cancers in the United States. Cancer Res.

[8] Chen H., He R., Shi X., Zhou M., Zhao C., Zhang H., Qin R. (2018). Meta-analysis on resected pancreatic cancer: a comparison between adjuvant treatments and gemcitabine alone. BMC Cancer.

[9] Hermann P.C., Sainz B. (2018). Pancreatic cancer stem cells: A state or an entity?. Semin. Cancer Biol.

[10] Tsai K.K., Chan T.S., Shaked Y. (2019). Next viable routes to targeting pancreatic cancer stemness: learning from clinical setbacks. J. Clin. Med.

[11] Cha Y., Erez T., Reynolds I.J., Kumar D., Ross J., Koytiger G., Kusko R., Zeskind B., Risso S., Kagan E., Papapetropoulos S., Grossman I., Laifenfeld D. (2018). Drug repurposing from the perspective of pharmaceutical companies. Br. J. Pharmacol.

[12] Gns H.S., Gr S., Murahari M., Krishnamurthy M. (2019). An update on drug repurposing: re-written saga of the drug's fate. Biomed. Pharmacother.

[13] Nowak-Sliwinska P., Scapozza L., Altaba A.R.I. (2019). Drug repurposing in oncology: compounds, pathways, phenotypes and computational approaches for colorectal cancer. Biochim. Biophys. Acta Rev. Cancer.

[14] Pulley J.M., Rhoads J.P., Jerome R.N., Challa A.P., Erreger K.B., Joly M.M., Lavieri R.R., Perry K.E., Zaleski N.M., Shirey-Rice J.K., Aronoff D.M. (2020). Using what we already have: uncovering new drug repurposing strategies in existing omics data. Annu. Rev. Pharmacol. Toxicol.

[15] Okada M., Kuramoto K., Takeda H., Watarai H., Sakaki H., Seino S., Seino M., Suzuki S., Kitanaka C. (2016). The novel JNK inhibitor AS602801 inhibits cancer stem cells *in vitro in vivo*. Oncotarget.

[16] Okada M., Takeda H., Sakaki H., Kuramoto K., Suzuki S., Sanomachi T., Togashi K., Seino S., Kitanaka C. (2017). Repositioning CEP-1347, a chemical agent originally developed for the treatment of Parkinson's disease, as an anti-cancer stem cell drug. Oncotarget.

[17] Sato A., Sunayama J., Okada M., Watanabe E., Seino S., Shibuya K., Suzuki K., Narita Y., Shibui S., Kayama T., Kitanaka C. (2012). Glioma-initiating cell elimination by metformin activation of FOXO3 via AMPK. Stem Cells Transl. Med.

[18] Sanomachi T., Suzuki S., Kuramoto K., Takeda H., Sakaki H., Togashi K., Seino S., Yoshioka T., Okada M., Kitanaka C. (2017). Olanzapine, an atypical antipsychotic, inhibits survivin expression and sensitizes cancer cells to chemotherapeutic agents. Anticancer Res.

[19] Suzuki S., Okada M., Kuramoto K., Takeda H., Sakaki H., Watarai H., Sanomachi T., Seino S., Yoshioka T., Kitanaka C. (2016). Aripiprazole, an antipsychotic and partial dopamine agonist, inhibits cancer stem cells and reverses chemoresistance. Anticancer Res.

[20] Suzuki S., Yamamoto M., Togashi K., Sanomachi T., Sugai A., Seino S., Yoshioka T., Kitanaka C., Okada M. (2019). *In vitro in vivo* anti-tumor effects of brexpiprazole, a newly-developed serotonin-dopamine activity modulator with an improved safety profile. Oncotarget.

[21] Keith B.D. (2008). Systematic review of the clinical effect of glucocorticoids on nonhematologic malignancy. BMC Cancer.

[22] Lien H.C., Lu Y.S., Shun C.T., Yao Y.T., Chang W.C., Cheng A.L. (2008). Differential expression of glucocorticoid receptor in carcinomas of the human digestive system. Histopathology.

[23] Maurice-Dror C., Perets R., Bar-Sela G. (2018). Glucocorticoids as an adjunct to oncologic treatment in solid malignancies—not an innocent bystander. Crit. Rev. Oncol. Hematol.

[24] Norgaard P., Poulsen H.S. (1991). Glucocorticoid receptors in human malignancies: a review. Ann. Oncol.

[25] Woenckhaus J., Franke F.E., Hackethal A., Von Georgi R., Münstedt K. (2006). Glucocorticosteroid receptors in ovarian carcinomas. Oncol. Rep.

[26] Avenant C., Ronacher K., Stubsrud E., Louw A., Hapgood J.P. (2010). Role of ligand-dependent GR phosphorylation and half-life in determination of ligand-specific transcriptional activity. Mol. Cell. Endocrinol.

[27] Wallace A.D., Cidlowski J.A. (2001). Proteasome-mediated glucocorticoid receptor degradation restricts transcriptional signaling by glucocorticoids. J. Biol. Chem.

[28] Okada M., Shibuya K., Sato A., Seino S., Suzuki S., Seino M., Kitanaka C. (2014). Targeting the K-Ras–JNK axis eliminates cancer stem-like cells and prevents pancreatic tumor formation. Oncotarget.

[29] Recio-Boiles A., Ilmer M., Rhea P.R., Kettlun C., Heinemann M.L., Ruetering J., Vykoukal J., Alt E. (2016). JNK pathway inhibition selectively primes pancreatic cancer stem cells to TRAIL-induced apoptosis without affecting the physiology of normal tissue resident stem cells. Oncotarget.

[30] Fang M., Li Y., Huang K., Qi S., Zhang J., Zgodzinski W., Majewski M., Wallner G., Gozdz S., Macek P., Kowalik A., Pasiarski M., Grywalska E., Vatan L., Nagarsheth N. (2017). IL33 promotes colon cancer cell stemness via JNK activation and macrophage recruitment. Cancer Res.

[31] Kitanaka C., Sato A., Okada M. (2013). JNK signaling in the control of the tumor-initiating capacity associated with cancer stem cells. Genes Cancer.

[32] Tong M., Fung T.M., Luk S.T., Ng K.Y., Lee T.K., Lin C.H., Yam J.W., Chan K.W., Ng F., Zheng B.J., Yuan Y.F., Xie D., Lo C.M., Man K., Guan X.Y. (2015). ANXA3/JNK signaling promotes self-renewal and tumor growth, and its blockade provides a therapeutic target for hepatocellular carcinoma. Stem Cell Rep.

[33] Xie X., Kaoud T.S., Edupuganti R., Zhang T., Kogawa T., Zhao Y., Chauhan G.B., Giannoukos D.N., Qi Y., Tripathy D., Wang J., Gray N.S., Dalby K.N., Bartholomeusz C., Ueno N.T. (2017). c-Jun N-terminal kinase promotes stem cell phenotype in triple-negative breast cancer through upregulation of Notch1 via activation of c-Jun. Oncogene.

[34] Zhao R., Yu Z., Li M., Zhou Y. (2019). Interleukin-33/ST2 signaling promotes hepatocellular carcinoma cell stemness expansion through activating c-Jun N-terminal kinase pathway. Am. J. Med. Sci.

[35] Grynberg K., Ma F.Y., Nikolic-Paterson D.J. (2017). The JNK signaling pathway in renal fibrosis. Front. Physiol.

[36] Ratman D., Vanden Berghe W., Dejager L., Libert C., Tavernier J., Beck I.M., De Bosscher K. (2013). How glucocorticoid receptors modulate the activity of other transcription factors: a scope beyond tethering. Mol. Cell. Endocrinol.

[37] Shipp L.E., Lee J.V., Yu C.Y., Pufall M., Zhang P., Scott D.K., Wang J.C. (2010). Transcriptional regulation of human dual specificity protein phosphatase 1 (DUSP1) gene by glucocorticoids. PLoS One.

[38] Tchen C.R., Martins J.R., Paktiawal N., Perelli R., Saklatvala J., Clark A.R. (2010). Glucocorticoid regulation of mouse and human dual specificity phosphatase 1 (DUSP1) genes: unusual cis-acting elements and unexpected evolutionary divergence. J. Biol. Chem.

[39] Liu L., Aleksandrowicz E., Schönsiegel F., Gröner D., Bauer N., Nwaeburu C.C., Zhao Z., Gladkich J., Hoppe-Tichy T., Yefenof E., Hackert T., Strobel O., Herr I. (2017). Dexamethasone mediates pancreatic cancer progression by glucocorticoid receptor, TGFβ and JNK/AP-1. Cell Death Dis.

[40] Kreso A., van Galen P., Pedley N.M., Lima-Fernandes E., Frelin C., Davis T., Cao L., Baiazitov R., Du W., Sydorenko N., Moon Y.C., Gibson L., Wang Y., Leung C., Iscove N.N. (2014). Self-renewal as a therapeutic target in human colorectal cancer. Nat. Med.

[41] Nunes T., Hamdan D., Leboeuf C., El Bouchtaoui M., Gapihan G., Nguyen T.T., Meles S., Angeli E., Ratajczak P., Lu H., Di Benedetto M., Bousquet G., Janin A. (2018). Targeting cancer stem cells to overcome chemoresistance. Int. J. Mol. Sci.

[42] Zhao J. (2016). Cancer stem cells and chemoresistance: the smartest survives the raid. Pharmacol. Ther.

[43] Takeda H., Okada M., Suzuki S., Kuramoto K., Sakaki H., Watarai H., Sanomachi T., Seino S., Yoshioka T., Kitanaka C. (2016). Rho-associated protein kinase (ROCK) inhibitors inhibit survivin expression and sensitize pancreatic cancer stem cells to gemcitabine. Anticancer Res.

[44] Oettle H., Neuhaus P., Hochhaus A., Hartmann J.T., Gellert K., Ridwelski K., Niedergethmann M., Zulke C., Fahlke J., Arning M.B., Sinn M., Hinke A., Riess H. (2013). Adjuvant chemotherapy with gemcitabine and long-term outcomes among patients with resected pancreatic cancer: the CONKO-001 randomized trial. JAMA.

[45] Kostaras X., Cusano F., Kline G.A., Roa W., Easaw J. (2014). Use of dexamethasone in patients with high-grade glioma: a clinical practice guideline. Curr. Oncol.

[46] Arrizabalaga O., Moreno-Cugnon L., Auzmendi-Iriarte J., Aldaz P., Ibanez de Caceres I., Garros-Regulez L., Moncho-Amor V., Torres-Bayona S., Pernía O., Pintado-Berninches L., Carrasco-Ramirez P., Cortes-Sempere M., Rosas R., Sanchez-Gomez P., Ruiz I. (2017). High expression of MKP1/DUSP1 counteracts glioma stem cell activity and mediates HDAC inhibitor response. Oncogenesis.

[47] Mills B.N., Albert G.P., Halterman M.W. (2017). Expression profiling of the MAP kinase phosphatase family reveals a role for DUSP1 in the glioblastoma stem cell niche. Cancer Microenviron.

[48] Rhim A.D., Mirek E.T., Aiello N.M., Maitra A., Bailey J.M., McAllister F., Reichert M., Beatty G.L., Rustgi A.K., Vonderheide R.H., Leach S.D., Stanger B.Z. (2012). EMT and dissemination precede pancreatic tumor formation. Cell.

[49] Mikosz C.A., Brickley D.R., Sharkey M.S., Moran T.W., Conzen S.D. (2001). Glucocorticoid receptor-mediated protection from apoptosis is associated with induction of the serine/threonine survival kinase gene, sgk-1. J. Biol. Chem.

[50] Stringer-Reasor E.M., Baker G.M., Skor M.N., Kocherginsky M., Lengyel E., Fleming G.F., Conzen S.D. (2015). Glucocorticoid receptor activation inhibits chemotherapy-induced cell death in high-grade serous ovarian carcinoma. Gynecol. Oncol.

[51] Xiao M., Li W. (2015). Recent advances on small-molecule survivin inhibitors. Curr. Med. Chem.

[52] Gross K.L., Oakley R.H., Scoltock A.B., Jewell C.M., Cidlowski J.A. (2011). Glucocorticoid receptor α isoform-selective regulation of antiapoptotic genes in osteosarcoma cells: a new mechanism for glucocorticoid resistance. Mol. Endocrinol.

[53] Reagan-Shaw S., Nihal M., Ahmad N. (2008). Dose translation from animal to human studies revisited. FASEB J.

[54] Aiello N.M., Bajor D.L., Norgard R.J., Sahmoud A., Bhagwat N., Pham M.N., Cornish T.C., Iacobuzio-Donahue C.A., Vonderheide R.H., Stanger B.Z. (2016). Metastatic progression is associated with dynamic changes in the local microenvironment. Nat. Commun.

[55] Waghray M., Yalamanchili M., di Magliano M.P., Simeone D.M. (2013). Deciphering the role of stroma in pancreatic cancer. Curr. Opin. Gastroenterol.

[56] Call T.R., Pace N.L., Thorup D.B., Maxfield D., Chortkoff B., Christensen J., Mulvihill S.J. (2015). Factors associated with improved survival after resection of pancreatic adenocarcinoma: a multivariable model. Anesthesiology.

[57] Sandini M., Ruscic K.J., Ferrone C.R., Warshaw A.L., Qadan M., Eikermann M., Lillemoe K.D., Fernández-Del Castillo C. (2018). Intraoperative dexamethasone decreases infectious complications after pancreaticoduodenectomy and is associated with long-term survival in pancreatic cancer. Ann. Surg. Oncol.

[58] Huang W.W., Zhu W.Z., Mu D.L., Ji X.Q., Nie X.L., Li X.Y., Wang D.X., Ma D. (2018). Perioperative management may improve long-term survival in patients after lung cancer surgery: a retrospective cohort study. Anesth. Analg.

[59] Obradović M.M.S., Hamelin B., Manevski N., Couto J.P., Sethi A., Coissieux M.M., Münst S., Okamoto R., Kohler H., Schmidt A., Bentires-Alj M. (2019). Glucocorticoids promote breast cancer metastasis. Nature.

[60] Elinav E., Nowarski R., Thaiss C.A., Hu B., Jin C., Flavell R.A. (2013). Inflammation-induced cancer: crosstalk between tumours, immune cells and microorganisms. Nat. Rev. Cancer.

[61] Grivennikov S.I., Greten F.R., Karin M. (2010). Immunity, inflammation, and cancer. Cell.

[62] Yeh D.W., Huang L.R., Chen Y.W., Huang C.F., Chuang T.H. (2016). Interplay between inflammation and stemness in cancer cells: the role of toll-like receptor signaling. J. Immunol. Res.

[63] Hoppstädter J., Ammit A.J. (2019). Role of dual-specificity phosphatase 1 in glucocorticoid-driven anti-inflammatory responses. Front. Immunol.

[64] Hausmann S., Kong B., Michalski C., Erkan M., Friess H. (2014). The role of inflammation in pancreatic cancer. Adv. Exp. Med. Biol.

[65] Yu L.X., Ling Y., Wang H.Y. (2018). Role of nonresolving inflammation in hepatocellular carcinoma development and progression. NPJ Precis. Oncol.

[66] Bubici C., Papa S. (2014). JNK signalling in cancer: in need of new, smarter therapeutic targets. Br. J. Pharmacol.

[67] Kim J.B., Park S.Y., Kim H.R., Ahn Y.H., Jee H.G., Lee J.H., Yu S.J., Lee H.S., Lee M., Yoon J.H., Kim Y.J. (2015). JNK signaling in hepatocarcinoma cells is associated with the side population upon treatment with anticancer drugs. Mol. Med. Rep.

[68] Yamada H., Yoshida T., Sakamoto H., Terada M., Sugimura T. (1986). Establishment of a human pancreatic adenocarcinoma cell line (PSN-1) with amplifications of both c-myc and activated c-Ki-ras by a point mutation. Biochem. Biophys. Res. Commun.

[69] Hamilton T.C., Winker M.A., Louie K.G., Batist G., Behrens B.C., Tsuruo T., Grotzinger K.R., McKoy W.M., Young R.C., Ozols R.F. (1985). Augmentation of adriamycin, melphalan, and cisplatin cytotoxicity in drug-resistant and -sensitive human ovarian carcinoma cell lines by buthionine sulfoximine mediated glutathione depletion. Biochem. Pharmacol.

[70] Okada M., Shibuya K., Sato A., Seino S., Watanabe E., Suzuki S., Seino M., Kitanaka C. (2013). Specific role of JNK in the maintenance of the tumor-initiating capacity of A549 human non-small cell lung cancer cells. Oncol. Rep.

[71] Seino M., Okada M., Shibuya K., Seino S., Suzuki S., Ohta T., Kurachi H., Kitanaka C. (2014). Requirement of JNK signaling for self-renewal and tumor-initiating capacity of ovarian cancer stem cells. Anticancer Res.

[72] Shibuya K., Okada M., Suzuki S., Seino M., Seino S., Takeda H., Kitanaka C. (2015). Targeting the facilitative glucose transporter GLUT1 inhibits the self-renewal and tumor-initiating capacity of cancer stem cells. Oncotarget.

[73] Suzuki S., Okada M., Shibuya K., Seino M., Sato A., Takeda H., Seino S., Yoshioka T., Kitanaka C. (2015). JNK suppression of chemotherapeutic agents-induced ROS confers chemoresistance on pancreatic cancer stem cells. Oncotarget.

[74] Okada M., Hozumi Y., Tanaka T., Suzuki Y., Yanagida M., Araki Y., Evangelisti C., Yagisawa H., Topham M.K., Martelli A.M., Goto K. (2012). DGKζ is degraded through the cytoplasmic ubiquitin-proteasome system under excitotoxic conditions, which causes neuronal apoptosis because of aberrant cell cycle reentry. Cell. Signal.

[75] Lei K., Nimnual A., Zong W.X., Kennedy N.J., Flavell R.A., Thompson C.B., Bar-Sagi D., Davis R.J. (2002). The Bax subfamily of Bcl2-related proteins is essential for apoptotic signal transduction by c-Jun NH_2_-terminal kinase. Mol. Cell. Biol.

[76] Lindgren G., Kjellen E., Wennerberg J., Ekblad L. (2016). Wound-healing factors can prime head and neck cancer cells to increase their tumor-forming capacity. Laryngoscope.

[77] Sousa C.M., Biancur D.E., Wang X., Halbrook C.J., Sherman M.H., Zhang L., Kremer D., Hwang R.F., Witkiewicz A.K., Ying H., Asara J.M., Evans R.M., Cantley L.C., Lyssiotis C.A., Kimmelman A.C. (2016). Pancreatic stellate cells support tumour metabolism through autophagic alanine secretion. Nature.

