# Supplementary material for: Therapeutic targeting of pancreatic cancer stem cells by dexamethasone modulation of the MKP-1–JNK axis
Source: J Biol Chem. 2021 Jan 13;295(52):18328–42. doi: 10.1074/jbc.RA120.015223 (PMC7939393; doi:10.1074/jbc.RA120.015223)
Supplement: Supplementary file 1 [file mmc1.pdf]

Supporting Information

Therapeutic targeting of pancreatic cancer stem cells by dexamethasone modulation of the MKP-1 - JNK axis

Shuhei Suzuki[1, 2, 6], Masashi Okada[1, 6], Tomomi Sanomachi[1, 2], Keita Togashi[1, 3], Shizuka Seino[1], Atsushi Sato[4], Masahiro Yamamoto[1], Chifumi Kitanaka[1, 5]

Departments of [1]Molecular Cancer Science, [2]Clinical Oncology, [3]Ophthalmology and Visual Sciences, and [4]Neurosurgery, Yamagata University School of Medicine, Yamagata, 990-9585, Japan

[5]Research Institute for Promotion of Medical Sciences, Yamagata University Faculty of Medicine, Yamagata, 990-9585, Japan

Running title: GR-MKP-1 -JNK axis in pancreatic cancer stem cells

Contents:

Supporting Figures S1-S12

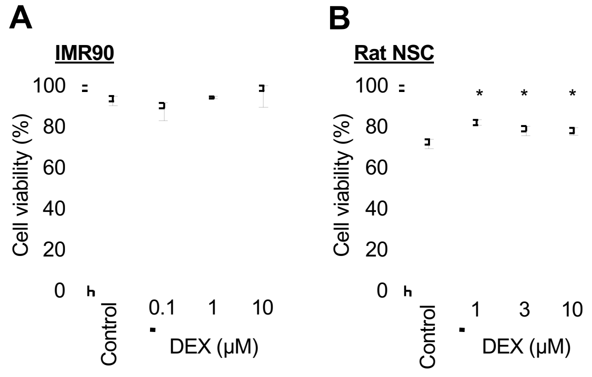

Supporting Figure S1. Dexamethasone effects on non-cancer cell viability. IMR90 human fetal lung fibroblasts (***A***) and rat cortical neural stem cells (Rat NSC) (***B***) either treated with DEX at the indicated concentrations or left untreated (Control) for six days were stained with trypan blue to determine cell viability. Data are shown as means ± standard deviation from triplicate samples of a representative experiment repeated with similar results. *$p < 0.05$ (compared with control).

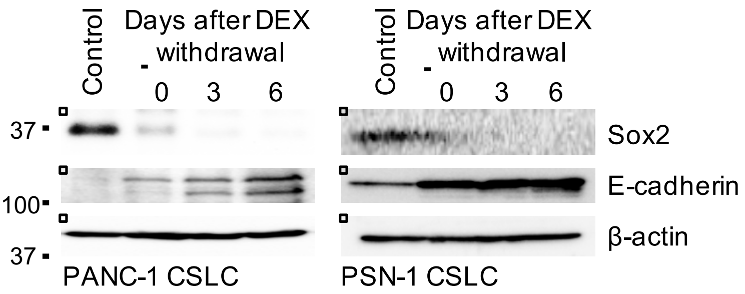

Supporting Figure S2. Dexamethasone-induced changes in differentiation and stem cell marker expression in pancreatic CSCs persist even after dexamethasone withdrawal. Cells maintained in either the presence or absence of 1 μM DEX for six days were subsequently cultured in DEX-free medium for the indicated time periods. Indicated proteins were detected by immunoblotting.

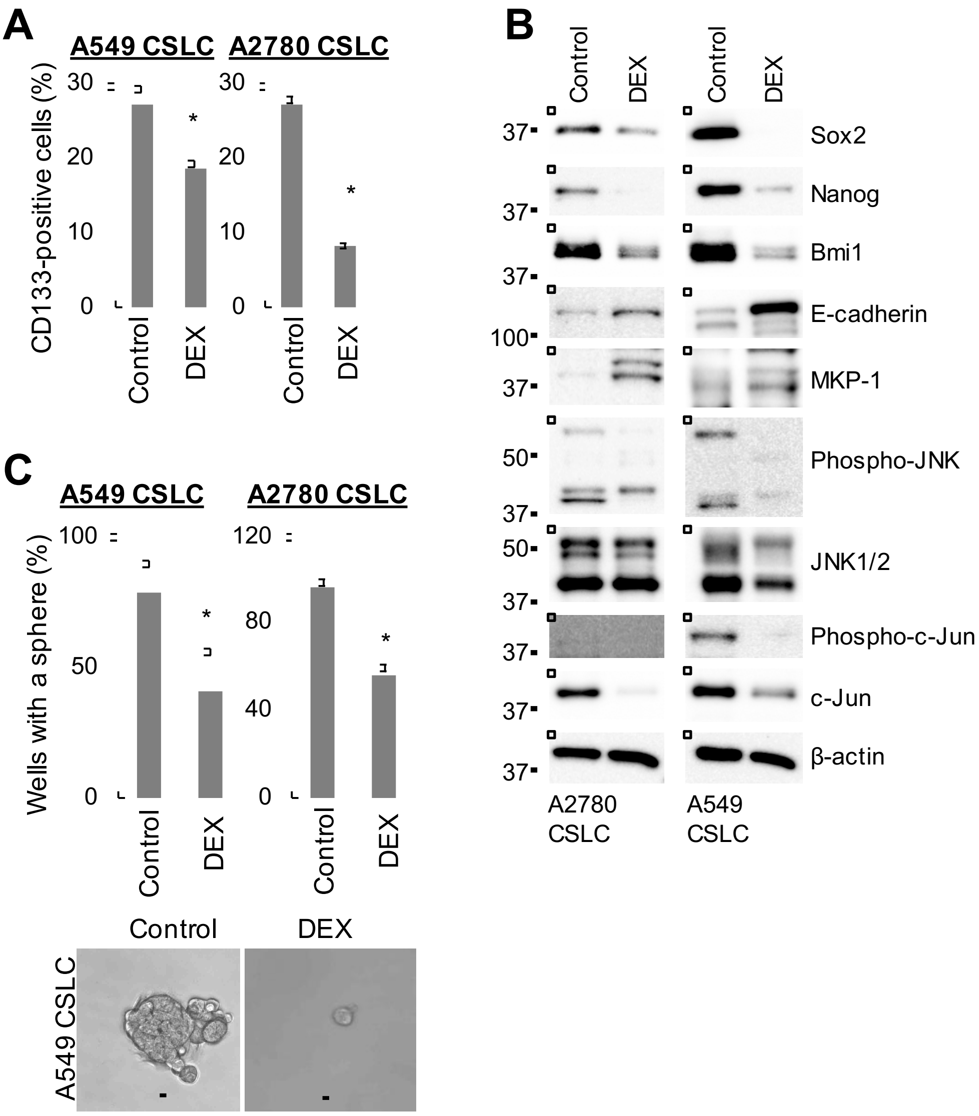

Supporting Figure S3. Dexamethasone promotes differentiation and loss of stemness in lung and ovarian CSCs. ***A***, A549 CSLC (non-small cell lung cancer) and A2780 CSLC (ovarian cancer) human cancer stem cells cultured either with 1 μM DEX or without drug (Control) for six days were subjected to flow cytometry to detect cell-surface expression of CD133; percentage of CD133-positive cells was determined. Data are shown as mean ±

standard deviation (SD) from three independent experiments (*$p < 0.05$). ***B*** and ***C***, Cells cultured as described in (***A***) were subjected to either immunoblotting for indicated proteins (***B***) or to a sphere formation assay in the absence of DEX (***C***). ***C***, Percentage of wells in which a sphere formed from a single cell is shown (*Top*). Data are shown as mean ± SD from three independent experiments. *$p < 0.05$. *Bottom*, photomicrographs of representative wells. Scale bars, 200 μm.

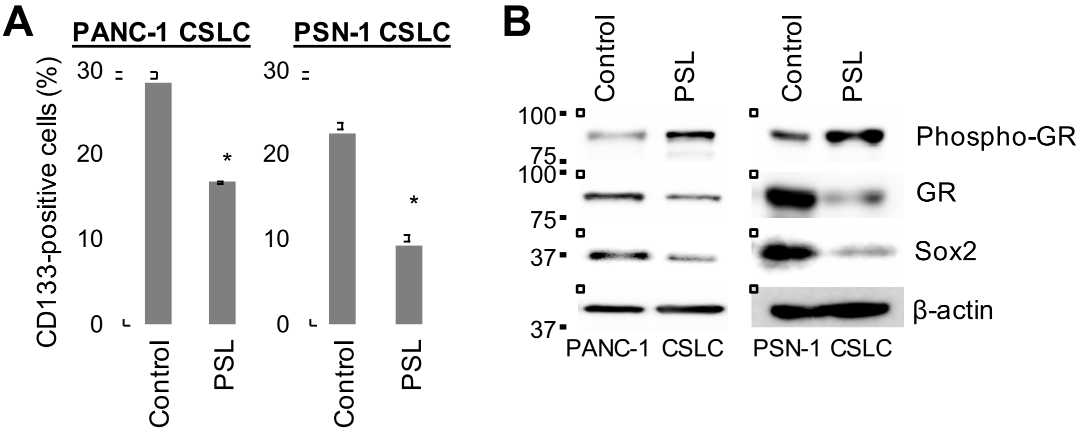

Supporting Figure S4. Prednisolone activates glucocorticoid receptor and promotes loss of stemness in pancreatic CSCs. ***A***, Cells cultured either with 1 μM prednisolone (PSL) or without PSL (Control) for six days were subjected to flow cytometry to detect cell-surface expression of CD133; percentage of CD133-positive cells was determined. Data are shown as mean ± standard deviation from three independent experiments. $^*p < 0.05$. ***B***, Cells cultured as described in (***A***) were subjected to immunoblotting for the indicated proteins. GR: glucocorticoid receptor.

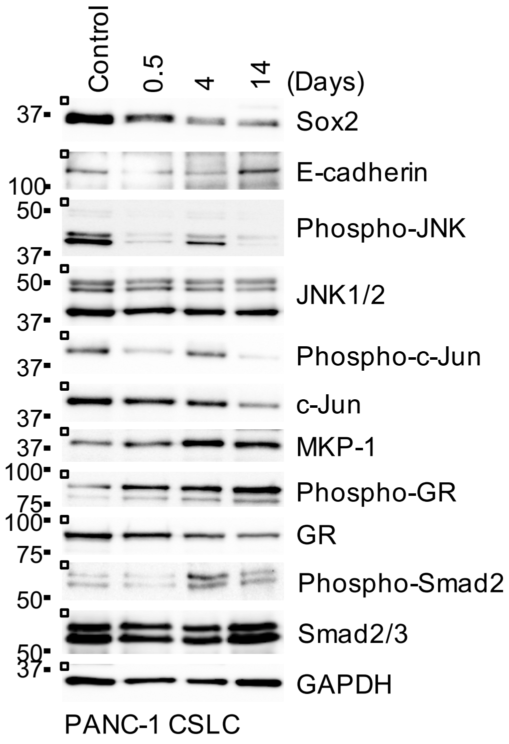

Supporting Figure S5. Multi-phasic change in JNK pathway activity after dexamethasone treatment. PANC-1 CSLC cells were cultured either with 1 μM DEXfor indicated times, or without DEX (Control) for 14 days. Indicated proteins were detected by immunoblotting. GR: glucocorticoid receptor.

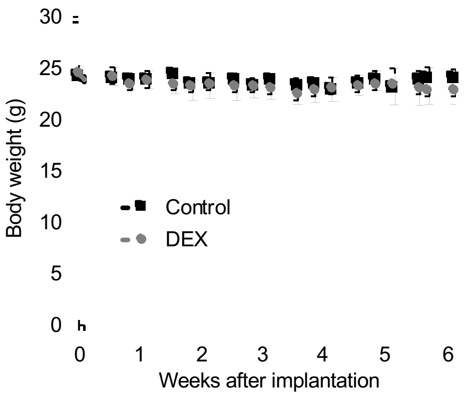

Supporting Figure S6. The effect of systemic dexamethasone administration on mouse body weight. Two groups of mice (n = 5 per group) were injected intraperitoneally with either vehicle only or 1 mg/kg DEX three times a week. Mouse body weight was measured at indicated time points. Data are shown as mean ± standard deviation for each treatment group.

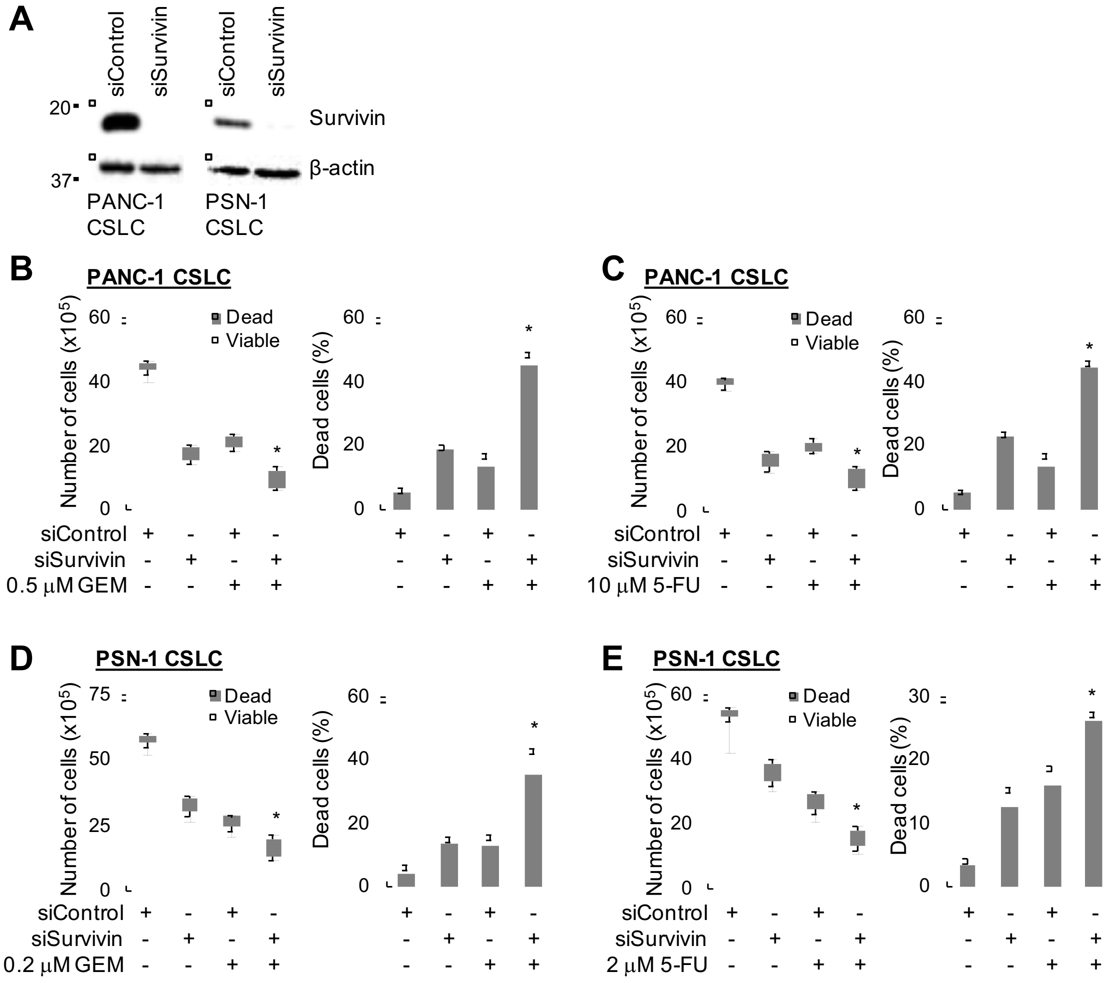

Supporting Figure S7. Role of survivin expression in chemoresistance of pancreatic CSCs. ***A***, Survivin levels were determined by immunoblotting in cells transiently transfected with either an siRNA against survivin (siSurvivin) or a control RNA (siControl). PANC-1 CSLC (***B***, ***C***) and PSN-1 CSLC (***D***, ***E***) cells transiently transfected with either siSurvivin or siControl were either treated on the day with the indicated concentrations of GEM or 5-FU or left untreated for three days. *Left*, Viable and dead cell numbers and *Right*, percentage of dead cells were determined by trypan blue staining. Data are shown as mean ± standard deviation from triplicate samples of a representative experiment. *$p < 0.05$.

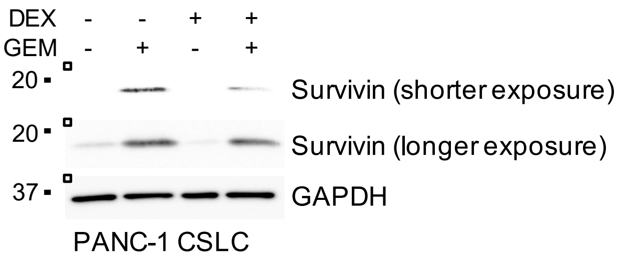

Supporting Figure S8. The effect of dexamethasone pretreatment on gemcitabine-induced survivin expression in pancreatic CSCs. PANC-1 CSLC cells pretreated with or without DEX (1 μM) for 6 days were treated with or without GEM (1 μM) for 3 days in the absence of DEX. Cells were then subjected to immunoblotting for survivin expression. The longer exposure panel is presented to show that the basal level of survivin expression is inhibited by DEX pretreatment.

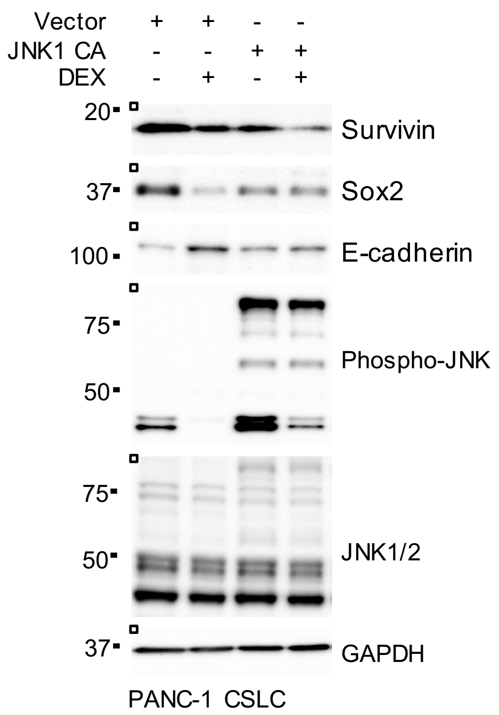

Supporting Figure S9. Forced JNK1 activation fails to prevent dexamethasone inhibition of survivin expression. PANC-1 CSLC cells transiently transfected with activated JNK1 protein expression plasmid (JNK1 CA) or with empty control vector (Vector) for 24 h were either treated with 1 μM DEX or left untreated for six days. Indicated proteins were detected by immunoblotting.

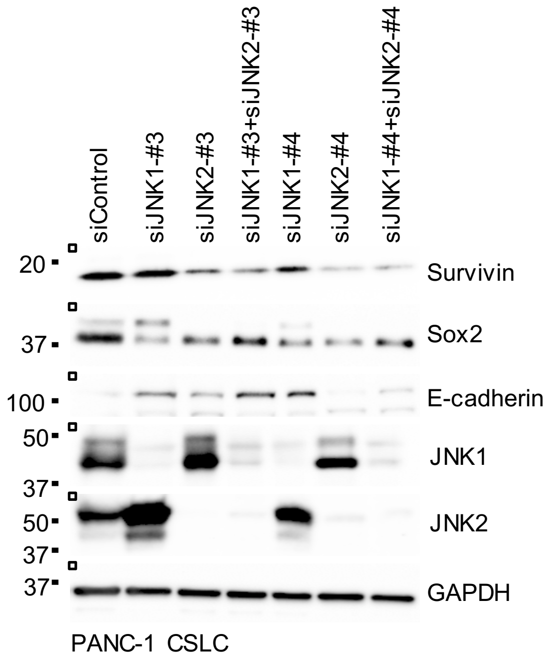

Supporting Figure S10. Differential requirement for JNK1 and JNK2 in the maintenance of stemness and survivin expression in pancreatic CSCs. PANC-1 CSLC cells were transiently transfected either with siRNA(s) against JNK1, JNK2, or both, or with a control RNA (siControl) for four days. Indicated proteins were detected by immunoblotting.

(Days)

		0	1	2	3	4	5	6	7	8	9	10	11	12	13	14	15	16	17	18	19	20
GEM (mg/kg)										40			40				40			40		
DEX (mg/kg)			1	1	1	1	1	1	1	1	1	0.5	0.5	0.33	0.33	0.25	0.25	[illegible]	[illegible]	0.125	0.125	0.1
Weeks		0								1							2					
Control	1	0								40			32				100			62.5		
	2	0								40			22.5				87.5			87.5		
	3	0								8			27				48			75		
	4	0								32			64				100			198		
	5	0								13.5			4				32			32		
	6	0								22.5			22.5				40			40		
GEM	1	0								27			27				75			87.5		
	2	0								13.5			48				75			87.5		
	3	0								6			56				64			56		
	4	0								18			48				75			87.5		
	5	0								22.5			75				40			75		
	6	0								22.5			18				40			40		
	7	0								27			13.5				32			40	?	
	8	0								22.5			18				75			40		
DEX+GEM	1	0								0			0.5				4			8		
	2	0								0			6				6			18		
	3	0								13.5			6				8			4		
	4	0								4			8				8			18		
	5	0								0			6				6			13.5		
	6	0								6			6				18			13.5		
	7	0								13.5			13.5				6			6		
	8	0								0			0				6			4		

		21	22	23	24	25	26	27	28	29	30	31	32	33	34	35	36	37	38	39	40
GEM (mg/kg)			40			40											40			40	
DEX (mg/kg)		0.1													1	1	0.5	0.25	0.125		
Weeks			3							4							5				
Control	1		108			126				196			256				221			288	
	2		87.5			144				220.5			220.5				270			352	
	3		87.5			108				126			171.5				196			320	
	4		198			180				198			245				270			320	
	5		40			40				108			75				88			108	
	6		48			48				108			108				172			126	
GEM	1		56			87.5				144			144				144			196	
	2		87.5			100				144			144				126			144	
	3		100			126				162			144				245			245	
	4		40			75				100			126				126			221	
	5		75			75				126			144				144			221	
	6		75			75				126			126				126			196	
	7		13.5			32				48			40				63			108	
	8		40			75				100			144				221			221	
DEX+GEM	1		6			6				18			13.5				18			18	
	2		8			4				4			13.5				18			18	
	3		6			6				13.5			13.5				32			32	
	4		18			4				13.5			6				18			32	
	5		13.5			13.5				6			18				40			63	
	6		13.5			8				18			40				56			88	
	7		4			6				18			13.5				14			18	
	8		0			6				13.5			18				32			18	

		41	42	43	44	45	46	47	48	49	50	51	52	53	54	55	56	57	58	59	60
GEM (mg/kg)				40			40				40			40							
DEX (mg/kg)		1	1	0.5	0.25	0.125			1	1	0.5	0.25	0.125								
Weeks				6							7							8			
Control	1			288			365				446			365							847
	2			486			384				567			384							847
	3			405			600				864			787							1372
	4			600			600				1008			1008							1183
	5			196			221				352			600							847
	6			162			162				352			567							1152
GEM	1			320			486				527			650							847
	2			221			288				405			600							600
	3			446			320				446			726							1008
	4			320			405				446			726							1008
	5			320			320				486			600							1008
	6			320			405				600			936							1690
	7			126			172				256			405							405
	8			288			288				352			486							650
DEX+GEM	1			18			32				75			108							196
	2			18			32				63			63							108
	3			40			40				14			32							14
	4			32			32				18			63							14
	5			108			108				126			108							63
	6			144			126				196			196							108
	7			14			14				18			6							4
	8			23			32				18			32							0

		61	62	63	64	65	66	67	68	69	70	71	72	73	74	75	76	77	78	79	80	81
GEM (mg/kg)					40			40				40			40				40			40
DEX (mg/kg)			1	1	0.5	0.25	0.125			1	1	0.5	0.25	0.125			1	1	0.5	0.25	0.125	
Weeks					9							10							11			
Control	1				1080			1352				1352			N/A				N/A			N/A
	2				968			1296				1296			N/A				N/A			N/A
	3				1568			1568				1800			1764				N/A			N/A
	4				1568			1764				1666			1862				N/A			N/A
	5				847			847				1183			1268				N/A			N/A
	6				1152			1437				1666			1764				N/A			N/A
GEM	1				1152			1152				1437			1521				N/A			N/A
	2				847			1080				1268			1568				N/A			N/A
	3				1470			1568				1913			N/A				N/A			N/A
	4				1268			1568				1913			N/A				N/A			N/A
	5				1268			N/A				N/A			N/A				N/A			N/A
	6				1960			N/A				N/A			N/A				N/A			N/A
	7				600			550				936			1008				1666			N/A
	8				847			908				1080			1352				1183			N/A
DEX+GEM	1				196			288				288			446				144			196
	2				108			172				108			196				196			500
	3				14			14				14			14				40			14
	4				4			14				4			6				0			14
	5				48			126				196			196				172			365
	6				144			256				365			365				108			63
	7				1			1				1			4				32			1
	8				6			8				18			18				32			18

Supporting Figure S11. Combination treatment with systemic dexamethasone and gemcitabine. Treatment schedule and effect on growth of tumors initiated by pancreatic CSCs are shown. Details of treatment schedule and tumor volume data from the mouse study shown in Fig. 8. Note that gray-shaded boxes indicate presence of subcutaneous tumor.

Supporting Figure S12

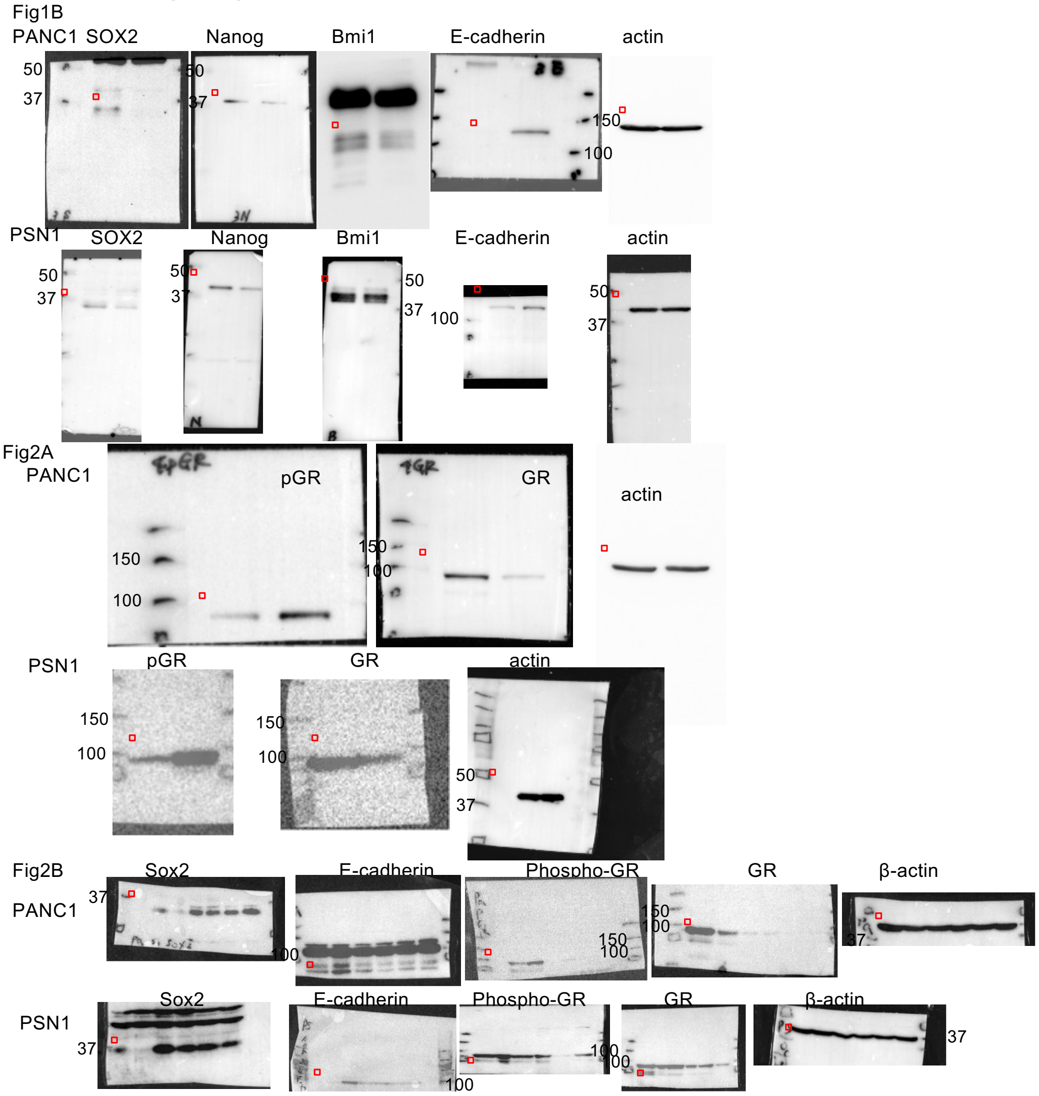

Supporting Figure S12 (continued)

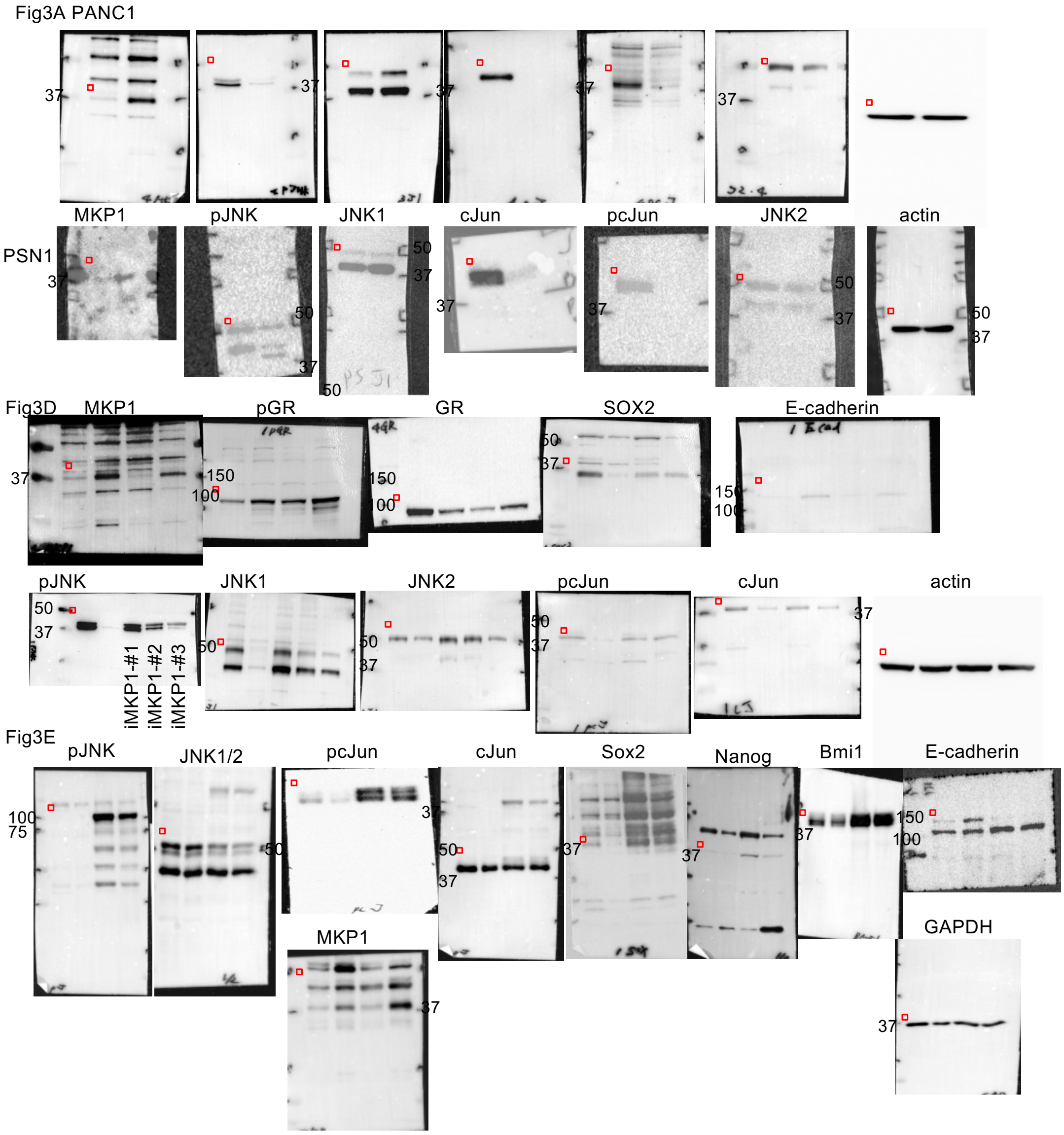

Supporting Figure S12 (continued)

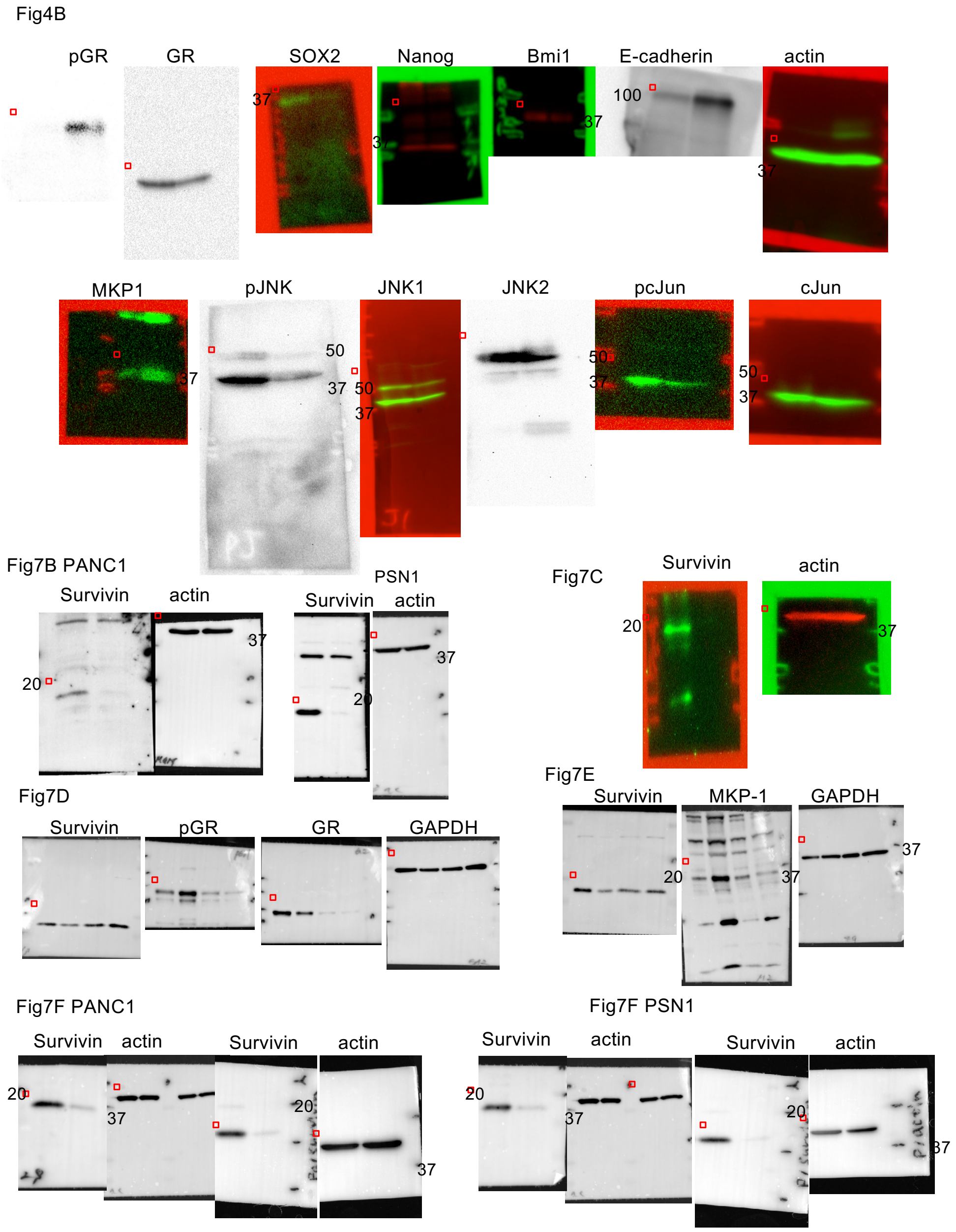

Supporting Figure S12 (continued)

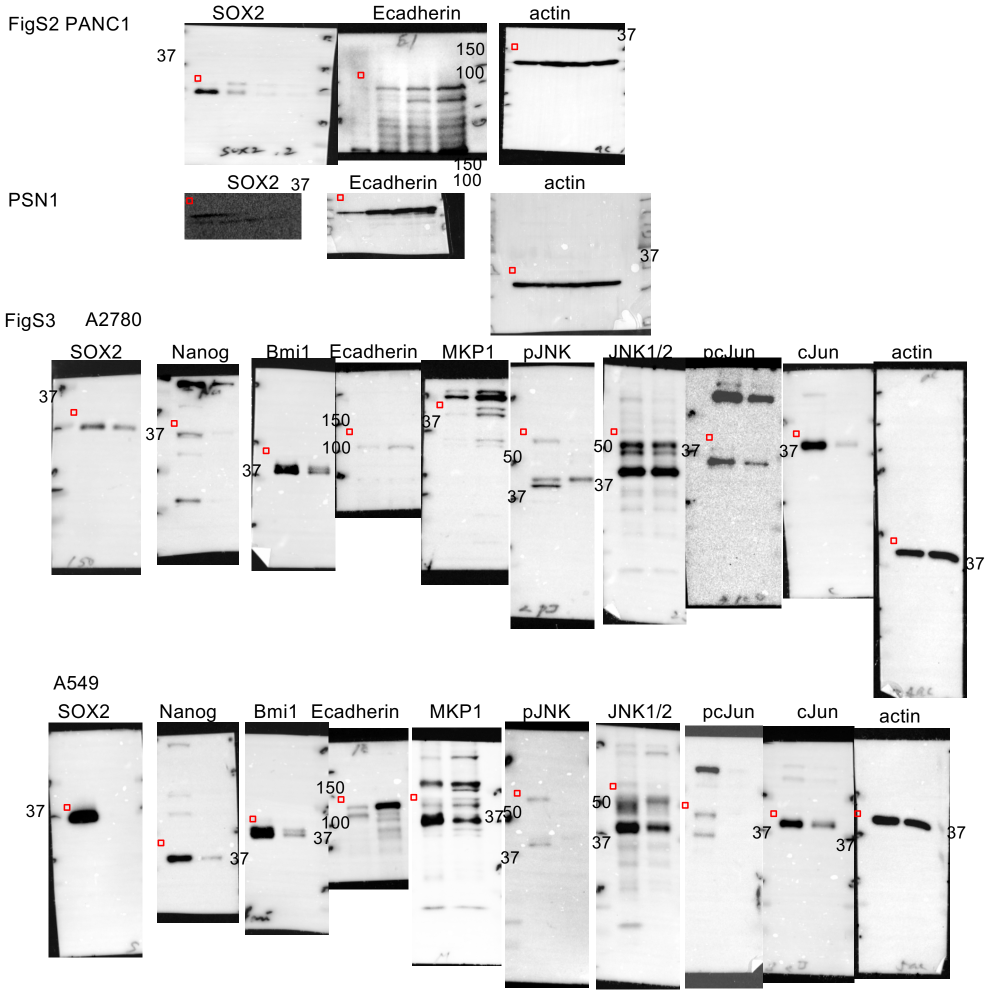

Supporting Figure S12 (continued)

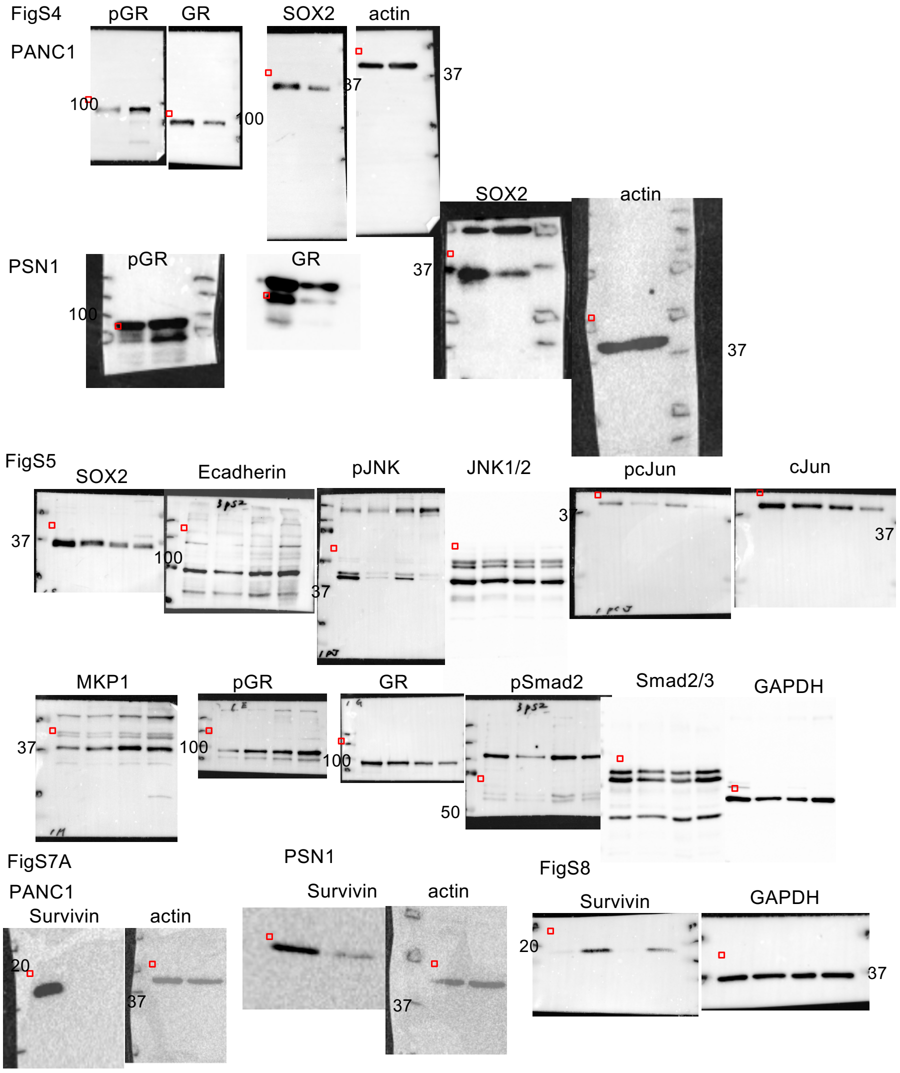

Supporting Figure S12 (continued)

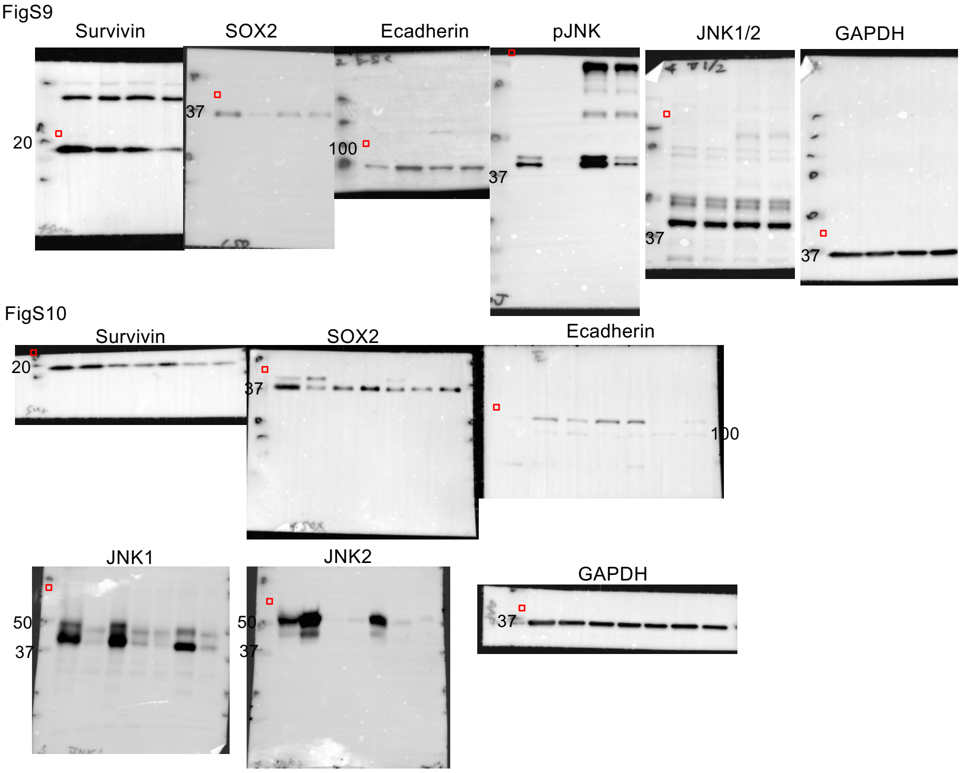

Supporting Figure S12. Unprocessed immunoblots. Unprocessed immunoblot images for Figs. 1B, 2A, 2B, 3A, 3D, 3E, 4B, 7B, 7C, 7D, 7E, 7F and for Supporting Figs. S2, S3, S4, S5, S7, S8, S9 and S10 are shown.
